# An Overview of Innovative Surface-Modification Routes for Pool Boiling Enhancement

**DOI:** 10.3390/mi15030302

**Published:** 2024-02-22

**Authors:** José Pereira, Reinaldo Souza, António Moreira, Ana Moita

**Affiliations:** 1 IN+ Center for Innovation, Technology and Policy Research, Instituto Superior Técnico, Universidade de Lisboa, Avenida Rovisco Pais, 1049-001 Lisboa, Portugal; reinaldo.souza@tecnico.ulisboa.pt (R.S.); aluismoreira@tecnico.ulisboa.pt (A.M.); anamoita@tecnico.ulisboa.pt (A.M.); 2 CINAMIL Centro de Investigação Desenvolvimento e Inovação da Academia Militar, Academia Militar, Instituto Universitário Militar, Rua Gomes Freire, 1169-203 Lisboa, Portugal

**Keywords:** pool boiling, enhanced surfaces, heat transfer, biphilic surfaces

## Abstract

This overview intends to provide a comprehensive assessment of the novel fluids and the current techniques for surface modification for pool boiling enhancement. The surface modification at macro-, micro-, and nanoscales is assessed concerning the underlying fluid routing and capability to eliminate the incipient boiling hysteresis and ameliorate the pool boiling heat-transfer ability, particularly when employed together with self-rewetting fluids and nanofluids with enriched thermophysical properties. Considering the nanofluids, it is viable to take the profit of their high thermal conductivity and their specific heat simultaneously and to produce a film of deposited nanoparticles onto the heating surface, which possesses enhanced surface roughness and an increased density of nucleation sites. Whilst the diverse improvement scales are found to achieve distinct levels of success regarding the nucleate boiling heat-transfer capability enhancement, it is also shown that the micro–nanoscale boiling surface features are susceptible to blockage, leading to the degradation of the improvement with time. Furthermore, topics relating to the heat transfer thermal behavior, ease of manufacture, cost-effectiveness, reliability, and durability are reviewed whenever available and challenges and recommendations for further research are highlighted.

## 1. Introduction

Surface coatings provide an efficient solution to modify boiling surfaces according to the demands of nucleate pool boiling heat transfer amelioration. Diverse coating methods have been employed for boiling heat-transfer enhancement, like sintering [1], spraying [2], etc., and distinct materials including metals, metal oxides, ceramics, polymers, and composites have been used for producing surface morphologies that include porous structures, pillar, bumps, channels, grooves, and pyramids. This modification affects different characteristics of the boiling heat transfer including the number of nucleation sites, roughness, wettability, and porosity. The characteristics of the surface, temperature, and pressure together with the working fluid thermophysical properties are the main factors for controlling the nucleate boiling heat-transfer performance. In the nucleate boiling heat transfer, the most relevant thermophysical characteristics of the operating fluid are the thermal conductivity, specific heat, viscosity, density, and surface tension, and the most prominent properties of the heating surface are the morphology, roughness, and wettability, characterized by contact angle measurements.

The modified surfaces for boiling heat transfer facilitate the nucleation and optimize the critical heat flux (CHF) by delaying the transition to the film boiling regime. For instance, the existing cavities on the heating surface originate bubbles and entrap them inside, and the surface roughness and the cavities together augment the number of active nucleation sites during the nucleate boiling regime. The surface-modification methods for the enhancement of the boiling heat-transfer capability can be mechanical, like sandblasting and laser machining [3]; alternatively, they can be surface-coating procedures, including physical vapor deposition, chemical vapor deposition, plasma, and spaying; there are also chemical processes, like oxidation and etching [4], and micro-electromechanical systems (MEMS) [5]. In the literature of the research field, the performance of any modified coated boiling surface can be evaluated through a comparison of its heat transfer coefficient (HTC) and CHF with those obtained using a bare boiling surface. Also, the exploration of innovative thermal fluids like the self-rewetting fluids and nanofluids are commonly employed methodologies for pool boiling heat transfer improvement.

Li et al. [6] have already published a review work regarding the techniques of passive and active pool boiling enhancement through surface modification. Although the survey of possible heat transfer amelioration approaches is comprehensive, the authors only covered passive modification by structured surfaces at a macro/microscale, coated-surface nanoparticles, nanowires, and other nanostructures, and heterogeneous wettability surfaces. The current review exhibits a wider range of surface modification techniques. This work still dedicated different sections to the passive modification routes, but it added innovative and updated methodologies; for instance, we have accounted for the very recent multiscale electroplated porous (MuSEP) coating for boiling surfaces. Also, more techniques are described herein, including detailed use of the displaced enhancement boiling attachments and the exploration of bi-conductive heat transfer surfaces. Apart from this, another novelty of the present work is the fact that it emphasizes the use of innovative fluids and additives like self-rewetting fluids, nanofluids, polymers, and surfactants. The combined usage of these materials with specific surface modification techniques brings new insights for pool boiling heat-transfer enhancement. Also, the mentioned authors did not present a dedicated section giving emphasis to certain parameters that directly influence the nucleate pool boiling heat-transfer enhancement; in contrast, the present work holds a section regarding the influence of the pressure of the system. All these factors equips the present overview to be one of the most complete and updated accumulations of information surrounding pool boiling enhancement techniques to date.

## 2. Surface Modification

### 2.1. Coating with Nanostructures

The accumulation of nanoparticles on the boiling surface can produce porous coatings that increase the heat transfer area, improve the wettability of the surface, and decrease its contact angle. Consequently, the enhancements in the wettability and heat transfer surface area induce a significant improvement in the nucleate boiling performance and CHF. In this direction Wu et al. [7] used titanium oxide and silica particles with a 10 µm diameter to spin-coat a copper surface, producing a hydrophilic boiling surface to infer the HTC and CHF for FC-72 and water. The authors observed that the hydrophilicity of the enhanced surface achieved considerable enhancements in CHFs of 38.2% and 50.4% for the FC-72 and water, respectively, in comparison to those obtained with an uncoated copper surface. Apart from being assembled onto surfaces, the nanoparticles can act as masking materials in conjunction with diverse etching methods to produce specific features in the modified surfaces. For instance, Chen et al. [8] used a self-masking methodology with nanoparticles as etching masks, released from a dummy material (cover glass) during the etching procedure to produce high-aspect-ratio nanoscale pillars of polymeric materials like polydimethylsiloxane. Moreover, Souza et al. [9] found that the deposition of 10 nm nanoparticles improved the HTC for HFE-7100, whilst 80 nm nanoparticles decreased the HTC as compared to a surface without nanoparticle deposition.

Regarding the use of porous boiling surfaces, Amaya et al. [10], concerning a potential application in pressurized water reactors, tested a nanoporous heat transfer surface in a pool boiling scenario of an aqueous solution of boric acid or borated water at 1% vol. The effect of system pressure and surface orientation on pool boiling heat transfer was studied. The nanoporous boiling surface was composed by a coating of alumina nanoparticles applied onto a plain copper surface with an area of 1 cm^2^ by the nanofluid boiling process. An uncoated surface was tested using borated water as the operating fluid, and because of the boric acid deposition, the boiling heat transfer deteriorated, and the CHF increased in reference to the one obtained with water. Additionally, the possibility of the transient pool boiling behavior presence using the borated water was examined, but this kind of behavior was not detected. Imposing orientation and operating pressure, the nanoporous surface using borated water showed a tendency based on further CHF enhancements to the CHF limit obtained by the nanoporous surface when using only water. Over the surface, the borated water CHF was increasingly improving with reducing working pressure in comparison with the one over the plain boiling surface. Nonetheless, the boiling heat transfer degraded slightly further. The authors attributed the boiling heat transfer degradation to the boric acid deposition onto the porous surface.

**Figure 1:**
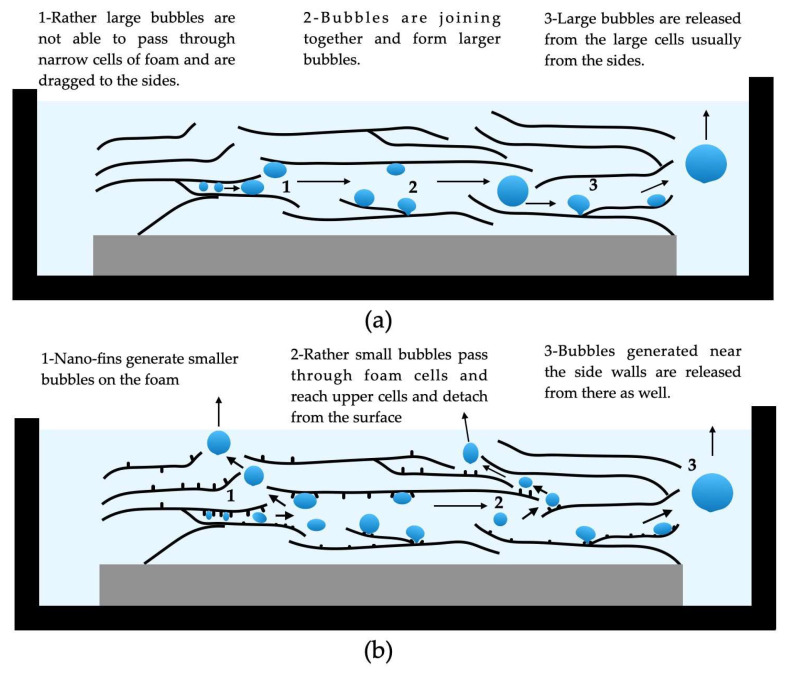
Mechanisms for bubble nucleation on a foam cover: (**a**) before coating with nanoparticles and (**b**) after coating with nanoparticles. Adapted from [11].

The nanoparticle deposition onto the heating surface is another possible way for heat-transfer enhancement. In this regard, Akbari et al. [11] evaluated the influence on the water pool boiling process of the deposition of silver nanoparticles onto porous copper foams. Two copper foams were employed: the CF1, with a porosity of 85% and 30 pores per inch, and the CF2, with a porosity of 90% and 40 pores per inch. These were welded on copper surfaces and coated with silver nanoparticles in water nanofluid, with a concentration of 25 mg/L. The results revealed that, when compared to a smooth copper heating surface, the uncoated CF1 and CF2 enhanced the HTC by 58% and 86% and decreased the wall superheat at onset nucleate boiling by 4.7 °C and 5.3 °C, respectively. Because of the generation of more and smaller bubbles and lower detachment frequencies, verified on the coated copper foams in respect to the uncoated foams, the deposited silver nanoparticles on CF1 and CF2 promoted further maximum HTC increases of 18% and 9%, respectively, and a further 0.3 °C reduction in the wall superheat at the onset of nucleate boiling. Figure 1 schematically represents the mechanisms for bubble generation on a foam cover before and after coating with nanoparticles.

Concerning the heat transfer benefits of using a heating surface with defects, Heitich et al. [12] determined the impact of nanostructured heat transfer surfaces on the water pool boiling under atmospheric pressure. The surfaces were composed of a constant tape substrate, which was nanostructured with molybdenum nanoparticles by the sputtering technique. During the pool boiling process with a maghemite nanofluid, the deposition of the maghemite nanoparticles onto the surfaces also occurred. The researchers concluded that the nanostructured surfaces showed higher wettability due to the greater population of surface defects produced by the nanoparticles. The surface defects in the material affect the contact angle and therefore may influence the heat transfer mechanisms and the CHF. The maghemite nanostructured surfaces showed greater porosity and roughness. These surfaces have a greater nanoparticle layer thickness and, consequently, a higher wettability compared to the molybdenum samples, suggesting that the roughness and thickness of the deposited layer also contributes to decreasing the contact angle. The samples with molybdenum-deposited nanoparticles showed a homogeneous distribution and hydrophilic characteristics. The rough surface exhibited a hydrophobic nature, while the other samples with nanoparticle deposition exhibited a hydrophilic nature. The nanostructures conducted to CHF increases, particularly in the case of the maghemite deposition, in which the CHF was approximately 139% higher than that of a plain heating surface. The CHF augmented with enhanced wettability. Enhancements in the CHF were reported, with decreasing apparent static and receding contact angles. The rough surfaces showed an HTC enhancement of nearly 19%, whereas other surfaces exhibited an enhancement in the boiling HTC at high-heat flux values. The surfaces with hydrophobic character promoted the HTC increase. The promising nucleation cavities were not flooded with the operating fluid, enabling vapor trapping, which contributed to the activation of the nucleation sites and, thus, to enhanced heat-transfer capability.

**Figure 2:**
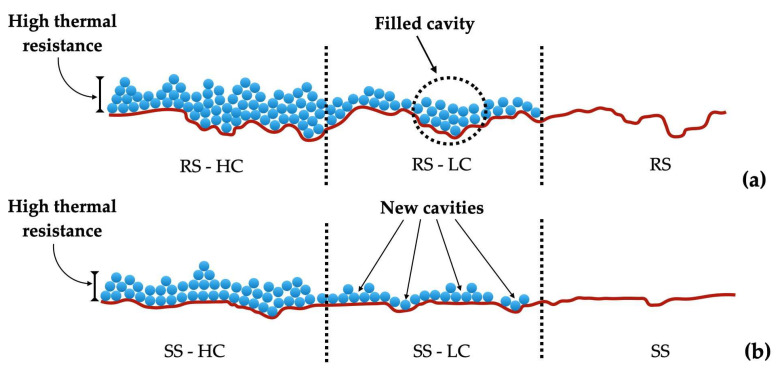
Schematic diagram of nanoparticles deposition on the (**a**) rough surface and (**b**) smooth surface. Adapted from [13].

The combined effect of the heating surface roughness and nanoparticle deposition on the nucleate boiling heat transfer was studied by the authors Kiyomura et al. [13], who prepared two rough surfaces coated with low and high mass concentrations of 0.029 g/L and 0.29 g/L, respectively, of iron oxide nanofluids produced by boiling, named RS-LC and RS-HC, respectively. Similarly, two other smooth surfaces were individually coated through low- and high-weight-fraction nanofluids, named SS-LC and SS-HC, respectively. The surfaces were then used for water pool boiling experiments. The obtained results for the water pool boiling showed that the RS-HC and RS-LC surfaces exhibited deterioration of the HTC in comparison with that of the originally rough boiling surface; in contrast, the HTC of the SS-LC was nearly 20% greater than those of the SS-HC and the original plain surface. After coating of the rough surfaces, the nucleation sites density was reduced and the thermal resistance increased as the greater surface cavities were filled with the nanoparticles of the nanofluid and, hence, smaller surface cavities were created. To ensure that the cavities remained active, a higher superheat degree was required; this was not beneficial for the boiling HTC. In contrast, after coating with nanoparticles, the smooth boiling surfaces exhibited an increased number of active nucleation sites. Nonetheless, an excessively thick nanoparticle coating augmented the thermal resistance of the boiling surface; therefore, the SS-LC surface presented better pool boiling heat-transfer characteristics. Figure 2 presents a schematic representation of the deposition of nanoparticles onto the rough and smooth surfaces.

Carbon nanotubes (CNT) are very thin tubes of graphitic carbon with typical outer diameters ranging from 1 to 100 nm and lengths between 1 and 50 µm. Carbon nanotubes have been chosen as a coating material for nucleate boiling enhancement purposes due to their high thermal conductivity and superior mechanical properties. The preparation of the carbon nanotubes relies fundamentally on techniques like electric arc discharge, laser ablation, and chemical vapor deposition (CVD) [14]. Other fabrication techniques are flame-based synthesis, electrolysis of molten halide salts, and cracking of hydrocarbons. Among all the production techniques, plasma-enhanced chemical vapor deposition (PECVD) is the most adequate technique to form aligned, individually standing, size-controlled carbon nanotubes. Furthermore, the wettability of a single carbon nanotube has been investigated extensively and it was concluded that materials with relatively low surface tension can wet the carbon nanotubes’ surface with contact angles lower than 90°. Also, a forest of vertically aligned carbon nanotubes grown and deposited on a polyacrylonitrile-based carbon fiber forms micro/nanoscale hierarchical surfaces; here, the carbon nanotubes reduce the contact area of a water droplet, inducing super-hydrophobicity.

The use of carbon nanotubes to enhance the pool boiling heat transfer was studied in the work conducted by Ahn et al. [15], who produced multi-wall carbon nanotubes by chemical vapor deposition on silicon heating surfaces to generate 9 µm tall and 25 µm tall carbon nanotubes in vertically aligned forests. The multi-walled carbon nanotubes had a diameter between 8 and 16 µm and a variable pitch between 8 nm and 16 nm. The heat transfer pool boiling performance achieved the same magnitude level for both heights of the nanotubes, but the taller nanotubes resulted in a 28% increment in the CHF as compared to only 25% with the shorter ones. This fact was interpreted since the taller nanotubes provided more suitable pathways for the liquid to flow to the active nucleation sites. The authors also explained the heat-transfer enhancement of the enhanced silicon during the film boiling regime as compared to plain silicon heating surfaces based on factors that include the high thermal conductivity of the carbon nanotubes, larger cold spots, enhanced ability to collapse the vapor film, improved liquid–solid contact area, and increased the heat transfer surface area. In the case of carbon nanotubes coatings on a metal substrate, the nanotubes are hydrophobic, enhancing the density of active nucleation sites and the bubbles generated on top of the carbon nanotubes coatings, whereas the untreated metal surface in their surroundings work as a hydrophilic region, delaying the film formation.

Also, regarding the use of nanotubes, Ujereh et al. [16] evaluated the nucleate boiling performance of silicon and copper heat transfer surfaces coated with CNT arrays, with different density and area coverage, by plasma-enhanced chemical vapor deposition. The researchers found that the silicon surface coated with the light CNT array was more capable of reducing the incipience superheat and increasing the HTC and CHF for the FC-72 refrigerant. The authors explained this evidence based on the ability of the CNT mesh to provide a high number of zero cone angles and cavities that were ripe for nucleation with minimal superheat. The authors reported that increasing the CNT mesh density on the silicon substrate slightly reduced the incipience superheat. However, very dense CNT arrays decreased CHF by reducing the effective heat transfer area as compared to the light CNT arrays. In addition, a higher heat-transfer enhancement was obtained with the CNT on silicon the surfaces than on the copper ones, given that the uncoated copper surfaces were rougher than the uncoated silicon surfaces, providing an abundance of active nucleation sites. Also, the titanium oxide nanotubes have been used by many researchers to properly modify heat transfer surfaces.

The use of nanotubes as boiling heat transfer surfaces was also examined by Wang et al. [17], who produced titanium oxide nanotubes surface arrays by electrochemical anodization of a plain titanium surface. The authors found that the wettability of the surface could be adjusted by ultra-violet irradiation, defining the photo-induced hydrophilic effect. The titanium oxide nanotube layer became super-hydrophilic when illuminated with ultra-violet light and slowly returned to the super-hydrophobic state when irradiated by visible light. The wettability transition was also verified in the case where the titanium oxide nanotube surface was modified with self-assembly monolayers of silanes and acids. This technique led to the decrement of the HTC but increased the CHF for water.

The exploration of hierarchical nanostructured heating surfaces has already been demonstrated to be a very suitable way of enhancing boiling heat transfer. In this sense, Wen et al. [18] synthesized a hierarchical boiling surface composed of long copper nanowires arrays surrounded by small copper nanowires; the specific arrangement of these induced the formation of microcavities between the short nanowire’s clusters. The authors reported an enhancement in the nucleate pool boiling performance for water, which was attributed mainly to the increased number of high-density nucleation sites, fluid rewetting by capillarity, and separation of fluid and vapor paths. The modified surface achieved 37% lower incipience superheat, 185% higher HTC, and 71% higher CHF as compared to a plain copper surface.

Overall, with the progress in micro/nano technologies, relevant surface modifications have been utilized to enhance pool boiling. Coated or thin-film-coated micro/nanosized particles offer another possible route for modifying the heat transfer surface. The surfaces coated with porous nanoparticles or thin films in advance can effectively achieve the dissipation of high-heat fluxes. There are several methodologies for the synthesis of coatings, including electrolytic deposition, plasma spraying, sintering, and galvanizing, among others. The roughness of the coated heat transfer surfaces is usually more pronounced; therefore, the density of the active nucleation sites and the capillary wicking are also enhanced. Coated surfaces include micro/nanoscale-particle-coated surfaces and surfaces which are coated by nanowires, nanofibers, and nanotubes. The approach to surface coating with micro/nanoparticles appears to be the most popular way of modifying surfaces due to the relatively simple process and its cost-efficiency. The micro/nanostructure layer surfaces can effectively enhance the boiling HTC and CHF caused by the enhanced surface roughness, the number of active nucleation sites, and the capillary wicking effect. Nonetheless, one of the main drawbacks of such surfaces is related to a relatively short lifespan, since the micro/nanoporous layers might peel off after several boiling cycles. Consequently, future research efforts should be made in the direction of refining and reinforcing these boiling surfaces to extend their boiling durability. In terms of nanowire-, nanofiber-, and nanotube-coated surfaces, the published works are still comparatively limited, and their cost of production is greater; hence, further in-depth research is strongly recommended. Also, when dealing with the deposition of nanoparticles during boiling, it seems that nanoparticle deposition mitigates some of the limitations that are usually posed by nanofluids, like sedimentation, agglomeration, precipitation, and surface degradation by erosion; these contribute to fluctuations in the heat-transfer performance over time. Nevertheless, more studies should be performed to better understand the deposition strength, robustness, durability, and homogeneity of nanoparticle deposition.

### 2.2. Porous Coatings and Structures

The microporous coatings for pool boiling heat-transfer enhancement purposes have intrinsic characteristics, like the increased heat transfer area, the enhanced density of active nucleation sites, the segregation of the liquid and vapor pathways, and the capillary wicking action; these greatly improve nucleate pool boiling performance. Microporous coatings can be produced by a wide range of techniques, like welding, sintering, electrolytic deposition, flame spraying, galvanizing, and polymer plasma spraying [19]. The main objective of the techniques is to synthesize a layer of potential nucleation cavities. The exploration of porous coatings as heat transfer surfaces was studied by Patil et al. [20]; the researchers used a two-step electrodeposition process to regulate the thickness and pore size of porous surface structures on copper substrates. The process entailed the application of high current density for a short period and a lower current density for an extended period. The lower-current-density cauliflower-like microstructures exhibited a heat-transfer performance that was superior to that achieved with the open-dish-like microstructures produced by higher current density, and the peak boiling HTC value was 176 kW/(m^2^·K).

The use of porous heating surfaces was investigated by Jun et al. [21]; the researchers assessed the pool boiling heat-transfer capability using microporous heating surfaces fabricated through brazing 25 µm copper particles coatings which were between 49 µm and 283 µm thick. Using water as an operating fluid, the authors identified three different heat transfer regimes: (i) the microporous regime, in which the HTC and CHF were augmented with the growing thickness of the coatings; (ii) the microporous–porous transition state, in which the CHF increased with the rising thickness of the coatings and the HTC was augmented at lower imposed heat fluxes and reduced at higher applied heat fluxes; (iii) the porous regime, in which the CHF and HTC decreased with the growing thickness of the coatings.

Another example is the work conducted by Rishi et al. [22], in which a porous coating composed of graphene nanoplatelets and copper was synthesized by a multistep electrodeposition procedure. In the procedure, the graphene nanoplatelets were incorporated into the electrolytic, which was deposited on the copper cathode during the electrodeposition process. The super-hydrophilic copper–graphene nanoplatelet coatings enhanced the CHF and HTC by 130% and 290%, respectively.

Under the same research scope, Zhou et al. [23] prepared a microporous copper boiling surface through single-step wire electric discharge machining. An HTC of 167 kW/(m^2^·K) and a CHF of 1857 kW/m^2^ were attained by taking advantage of the great number of microscale cavities induced by the manufacturing process; these were 198% and 61% greater than those obtained with a plain heating surface, respectively. The authors attributed the improved pool boiling response to the enhanced surface roughness and receding contact angle.

The boiling heat-transfer performance was also examined by Wang et al. [24]; the authors demonstrated that the micro–nanoporous copper heating surface, prepared through lower current density, can considerably improve the pool boiling heat-transfer ability. At 900 kW/m^2^ imposed heat flux, the boiling HTC of the developed surface was 1.7 times larger than that achieved with the original micro–nanoporous surface produced by the electrodeposition process and 4.8 times greater than that obtained with a smooth heat transfer surface.

**Figure 3:**
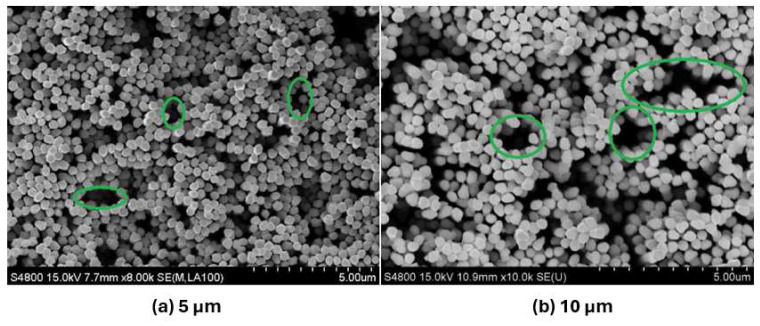
SEM top view of the defects in the nanowire arrays [25].

Shi et al. [25] synthesized copper nanowire arrays on copper surfaces via porous alumina membrane template-assisted electrodeposition. The copper nanowire arrays, presenting defects, improved the wettability and enhanced the nucleation site density; thus, they attained an improved nucleate boiling heat-transfer performance. Figure 3 displays SEM images of the defects in the nanowire arrays.

Still within the porous heating surfaces research topic, Kim et al. [26] studied the pool boiling heat transfer of microporous coatings for FC-72 and verified that, at heat fluxes inferior to 120 kW/m^2^, the dormant nucleation sites’ activation contributed to a larger share of latent heat in the process of heat transfer. Also, at heat fluxes superior to 120 kW/m^2^, a decrease in the amount of superheated fluid on the boiling surface occurred; this also reduced the contribution of the latent heat to the boiling heat transfer process.

**Figure 4:**
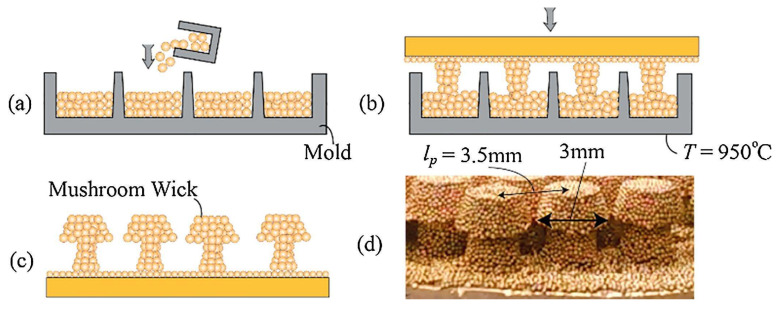
Schematic diagram of the manufacturing process on the mushroom-shaped wick by multi-step sintering. (**a**) Pouring of the mushroom-shaped cap particles into the second mold; (**b**) alignment of the columnar posts wick on the top upon mechanical pressure; (**c**) the mushroom-shaped posts’ wicks were released from the second mold; (**d**) manufactured mushroom-shaped posts’ wicks with *lp* of pitch [27].

Concerning the use of specifically shaped porous surface structures, Nasersharifi et al. [27] manufactured monolayer, columnar, and mushroom-shaped post wicks. A mushroom-shaped porous structure was produced via multi-step sintering; the pre-sintered columnar porous structure was positioned in the mold upside down, and the mushroom-shaped structure was produced on the columnar surface after the next sintering procedure. Also, the multilevel modulated wicks enhanced the HTC and CHF by regulating the fluid–vapor flow. The monolayer wicks with and without the mushroom-shaped post structure provided 87% and 20% CHF increases, respectively, in comparison to those obtained with a plain boiling surface. The improved pool boiling ability was attributed by the authors to the presence of the mushroom-shaped posts that decreased the hydrodynamic instability wavelength. Figure 4 schematically illustrates the fabrication of the mushroom wick through two-step sintering.

**Figure 5:**
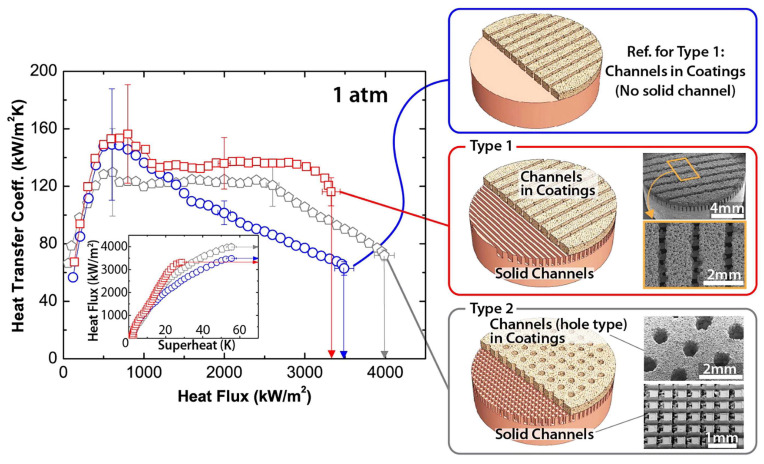
Hierarchical HTC enhancement structures at high-heat fluxes during pool boiling tests with water under 1 atm pressure [29].

The combined usage of porous coatings and surface structures for pool enhancement purposes has already been reported [28]. In particular, the combined usage of porous coatings and channels was evaluated by Ha et al. [29]; the researchers prepared copper microporous coatings with vapor channels. As can be seen in Figure 5, the 350 μm wide vapor channels were produced onto the microporous coating, employing a dicing saw that removed the particles, instead of cutting the surface structures. Also, the copper substrate and microporous layer had channels of vapor. The solid channels were produced using micro-milling; a two-step sintering process was adopted to prepare the improved structures. The parametric experimental investigation revealed that the HTC and CHF increased with growing channel depth and decreasing channel spacing. Figure 5 presents the hierarchical structures for HTC enhancement at high-heat fluxes.

The exploration of different porous and nonporous hydrophilic surfaces was studied by the researchers Seo et al. [30], who compared the CHF values using FC-72 and diverse hydrophilic boiling surfaces: (i) nonporous graphene deposition layer heating surface; (ii) nonporous silicon carbide deposition layer surface; (iii) porous graphene deposition layer surface; (iv) porous silicon carbide deposition layer surface. The substrate was a plain indium tin oxide surface; compared to this, the CHF for the different tested surfaces were, respectively, 9%, 16%, 90%, and 58% higher. The different values can be explained based on the heat-dissipation capability that was determined by the thermophysical characteristics of graphene and silicon carbide and on the hydrodynamic and capillarity derived from the surface porous structure and morphology.

Also, El-Genk and Ali [31,32] evaluated the pool boiling thermal behavior for the PF-5060 refrigerant and microporous copper heating surfaces produced by two-stage electrodeposition and reported an increment of the CHF with growing thickness of the porous layer from 80 µm to 230 µm. The first electrodeposition stage, in which a current with a density of 3 A/cm^2^ was applied, formed a high-porosity surface, comprising more than 90% pores; these were in a regular arrangement of open macropores, which were surrounded by branching and fine–dense dendrites. The second deposition stage gave more strength to the microstructure by further electrochemical deposition with considerably smaller current density during extended time. During the second deposition stage, microparticle clusters masked the dendrite microstructure. The alterations were reflected in a porosity between 65% and 80% and in an enhanced wetted surface area.

**Figure 6:**
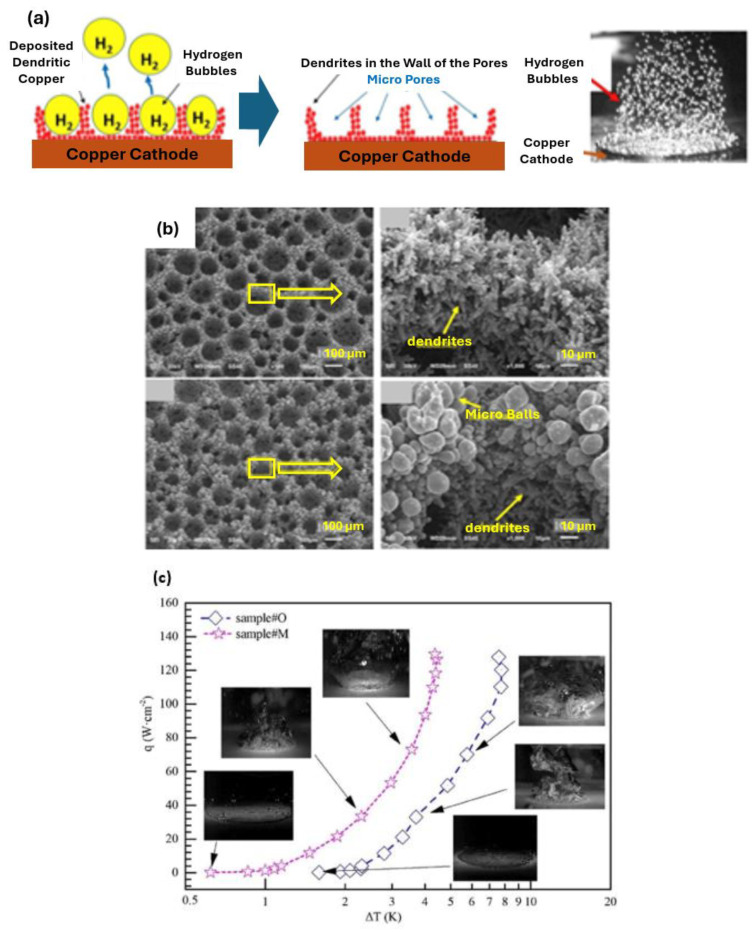
(**a**) Schematic diagram of micro–nanoscale biporous copper boiling Surface 1 synthesis. (**b**) SEM image of Surface 1 and 2. (**c**) Images taken from the bubble dynamic and plot of the heat flux against wall superheat [33].

The exploration of hierarchical porous boiling surfaces was analyzed by Wang et al. [33]; the researchers synthesized two micro–nano hierarchical porous copper boiling surfaces. Surface 1 was produced at a high current density. On the basis of Surface 1, a smaller current density was applied to prepare Surface 2. The large pore size of the heat transfer surfaces was approximately 100 μm. Moreover, the nanoscale dendrite on Surface 2 was extended to micro balls because of the lower current density. It was expected that the increasing size of the particles induced the reduction in the surface energy and, consequently, the vapor bubble detachment was strongly facilitated. The authors confirmed that Surface 2 possessed a stronger wickability than Surface 1, and that the wickability effect of the porous surface structures could undoubtedly improve the CHF, despite it having only a minor effect on the HTC values. The experimental results indicated that both samples exhibited similar increases in the CHF. The reason behind that fact was that the Surface 2 possessed a smaller surface energy or, in other words, a lower wettable capability, which was not beneficial for the CHF. Considering the benefits coming from the greater wickability, the combined effect was that the two heating surfaces exhibited similar CHF values. The HTC of Surface 2 was of 300 kW/(m^2^·K) at CHF, which was higher than the one obtained using Surface 1 and a plain boiling surface. Additionally, the authors found that the micro–nano biporous boiling surfaces decreased the period of the vapor bubble growth stage in respect to that verified using monotonous microstructure or nanostructure. Figure 6 summarizes the main findings of the experimental work.

Overall, the main conclusion is the fact that the porous structures in the boiling surfaces improve the spreading of the fluid under the vapor conglomerates, conducting to the rewetting of the dry regions in the pre-crisis mode and a CHF enhancement during the pool boiling process. Also, the nanoporous structures offer many challenges for their use in pool boiling. One of them is the preservation of the surface nanostructure over time; considering the alterations in the heat-transfer performance in the already performed extended lasting tests, it is vital to assess the heat transfer surfaces for any modifications in the surface nanostructure in time. Moreover, it is possible to form, at the same time, nanostructures (e.g., rods and fiber arrays) and porous structures using templates with nanopores. The template nanopores are dense, uniform, and offer the ideal tool to produce a high yield of nanostructures like, for instance, nanorod arrays [34]. Further template removal forms a porous network, which is a template replica. One practical example is the case of the synthesis of polyacrylonitrile along with a template of an anodized aluminum oxide porous membrane, which has been employed to form aligned polyacrylonitrile nanofiber arrays and demonstrated to possess super-hydrophobicity [35].

### 2.3. Multi-Scale Electroplated Porous (MuSEP) Coating

MuSEP coating [36,37,38,39,40,41] is a multi-scale porous electroplated coating which is capable of increasing the pool boiling efficiency in the cases where the coating is configured as the heat transport surface for the immersion cooling of a data processor. Some of its beneficial features are the increased HTC, the promotion of boiling incipience, and the delaying of CHF. This coating can be characterized as comprising a plurality of metal-based grain-defining pores, where the pores form more than 35% of void area. Also, the coating can be further characterized by having a pore-size gradient along at least one dimension of the coating. Another beneficial characteristic of MuSEP coating is the diversity of pore sizes and the existence of larger grains on the top layer. MuSEP coating delivers larger pores than the high thermal dissipation power in new-generation processors, which is excessively demanding for cooling systems. Immersion cooling using the phase-change of dielectric liquids is a viable candidate for electronic cooling. Porous coatings are one of the most efficient methods of increasing the boiling heat transfer and evacuating heat from electronic components under immersion cooling.

MuSEP coatings can be compared to the commercially available boiling enhancement coating BEC^®^, from 3M Corporation, which has an enhanced boiling performance and a low fabrication cost. For example, the number of pores within the size of more than 200 microns on both coatings was measured. The MuSEP coating had approximately four times more pores than the BEC^®^. Furthermore, the grain size increases along the thickness of the coating; smaller grains exist at the bottom of the MuSEP coating. These small grains provide smaller pores near the surface, where the bubbles are nucleated. Also, the larger grains at the top layer could serve as a liquid-wicking structure. On the contrary, the BEC^®^ has a more uniform texture with smaller pores, delivering a narrower pore size distribution and lacking a proper wicking structure. The MuSEP coating can be produced by electroplating deposition of porous copper onto a copper integrated heat spreader. The deposition can be done according to four different steps in which the current density and the duration of plating are varied.

The porous coating structure can be fabricated by a first electroplating step performed to obtain an anchor porous layer on the substrate layer, where there was proper adhesion made between the anchor porous layer and the substrate layer underneath the porous layer. A low current density of approximately 30 mA/cm^2^ was applied at this step for 5 min. A second electroplating stage was performed to obtain a highly porous layer for providing enough active sites for grains nucleation. A high current density between 500 and 600 mA/cm^2^ was applied at this step for 10 s, showing dendrite-shaped structures that formed a porous structure on the top of the substrate. A third electroplating step was performed to further form grains on the top of the previous porous structure. A medium current density of 150 mA/cm^2^ was applied for 10 min at this step. The second porous layer completely disappeared at this step, and a new porous structure was formed. The dendrite-shaped structures from the second electroplating exhibited a certain degree of growth and fortification. A fourth electroplating step was performed to make the structures of the porous coating more rigid. A low current density of 30 mA/cm^2^ was applied for 3 to 5 h at this step. On the other hand, smaller grains exist at the bottom of the MuSEP coating. These small grains provide smaller pores near the surface, where the bubbles are nucleated. On the other hand, larger grains at the top layer could serve as a liquid-wicking structure.

On the contrary, the BEC^®^ has a more uniform texture with smaller pores, delivering a narrower pore size distribution and lacking a proper wicking structure. The bare heating surface reached the CHF at 284 W, with an applied heat flux of 128 kW/m^2^, while the CHF is not reached for the porous surfaces up to 490 W. At a medium power, like 250 W, which is in the range of the TDP of new powerful microprocessors, the surface temperature of the bare surface was 92 °C; meanwhile, it was 79 °C for the BEC^®^ and 68 °C for the MuSEP coating. By comparing the BEC^®^ and the MuSEP coating, it is beneficial to have a multi-layered porous structure with multiple pore sizes and larger spaces for bubbles to escape. Without the intent of being bound by a specific theory, the inventors believe that, at low power, tiny pores are active and deliver small bubbles. By raising the input power, the growth of the bubbles is faster and more bubbles with larger sizes depart from the surface. It is also likely that bubbles coalesce underneath the porous structure and make even larger bubbles.

Larger bubbles need more space to travel upward. Therefore, larger pores provide more space at higher power levels for bubble growth and wider channels for bubbles to escape. The morphology of the MuSEP coating is such that it generates large void spaces (with an average size of at least 100 µm) between the grains at the surface of the MuSEP coating. The bubbles are mostly generated in these void spaces. At high powers, the bubbles will grow faster, and the delay between each departure is lowered, leading to the formation of bubble columns. It is always favorable that the vapor bubble columns do not interact with each other to avoid their merging. In addition to wicking the liquid to the boiling spots, the large grains separate the bubble columns, which help in delaying the CHF.

For the MuSEP coating, the increasing trend was even sharper, which shows the advantage of the MuSEP coating even at low powers. For example, at 60 W, with an imposed heat flux of 270 kW/m^2^, the BEC^®^ showed a 128% enhancement in comparison to that of the bare copper surface; in contrast, the MuSEP coating improved the HTC by 163%. The increasing trend continued for the MuSEP coating until it reached a peak boiling HTC value of 52 kW/m^2^ K at around 307 W. This superior performance could be related to the multi-scale pore structure and the possibility of generating more nucleation sites at a higher power range. Having numerous multi-scale pore sizes allows the surface to function more efficiently over a broader range of applied heat fluxes. The porosity of the MuSEP varies from small pores at the bottom to large pores at the top for the MuSEP coating. It has been reported that a surface generating smaller bubbles at a higher frequency is more efficient for heat transfer. There is a significant improvement in cooling performance when the MuSEP coating is applied on the surfaces compared to boiling on the bare surface.

Boiling on the MuSEP-coated surface decreases the junction temperature by 20 °C at different power levels compared to boiling on the bare surface. Comparing the two porous-coated boiling scenarios indicates that boiling directly on the die is more efficient at low powers, lower than 70 W. At powers higher than 80 W, the coated integrated heat spreader had a superior performance, which emphasizes the advantages of having a heat spreader in the CPU packaging, providing a larger surface area for boiling. An example of such technological heat transfer solutions is the work conducted by Larimi et al. [39], who developed an MuSEP coating for removing heat from electronic components under immersion cooling.

**Figure 7:**
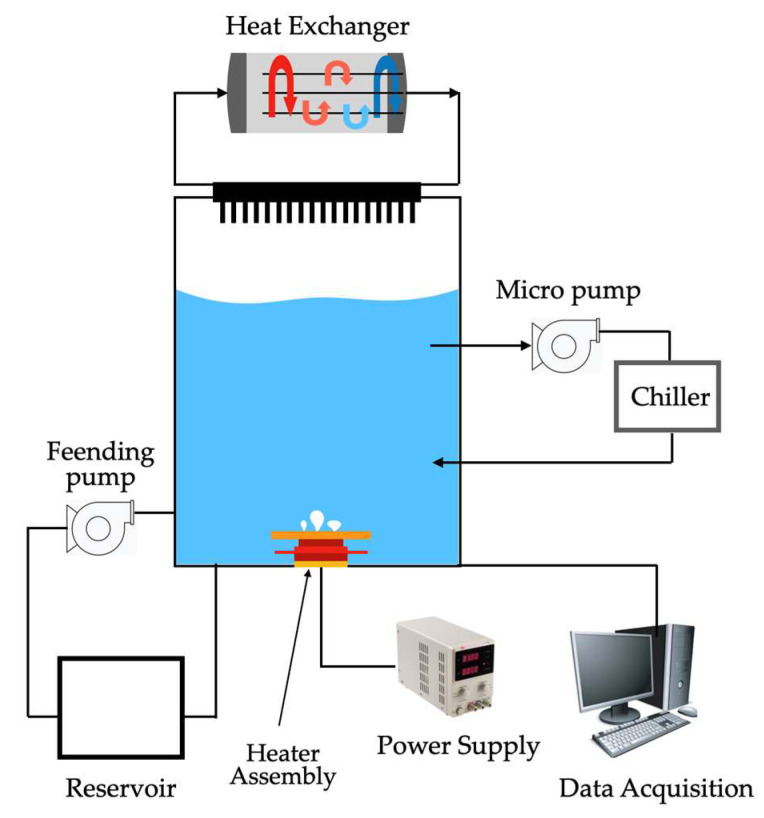
Schematic representation of the pool boiling setup. Adapted from [39].

**Figure 8:**
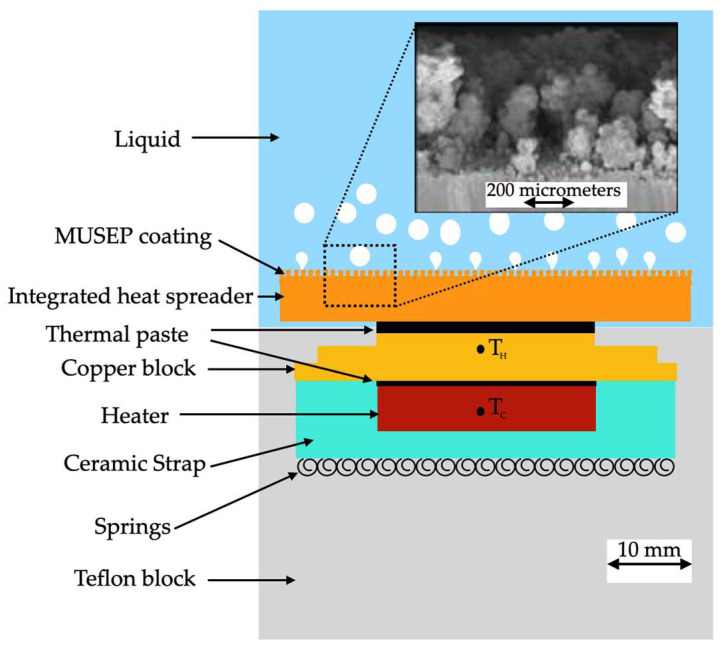
Schematic illustration of the heater assembly with the side view of the MuSEP coating. Tc is the case temperature and TH is the temperature of the heater. Adapted from [39].

The coating was deposited at room temperature and could be included in off-the-shelf electronic parts like CPUs and GPUs. The commercial heat-transfer fluid Novec^®^ 649 was used in pool boiling experiments along with different heating surfaces: bare copper surface, BEC^®^ surface, and MuSEP coating surface. A 4 mm thick heat spreader, with an area of 22 cm^2^, was attached to a heater. The best results were achieved with the MuSEP coating, as it could improve the boiling HTC by 108% versus the bare copper surface and by 38% versus the BEC^®^ at 250 W, with an imposed heat flux through the boiling surface of 113 kW/m^2^. At that power, the case temperature was 68 °C for the MuSEP coating, 79 °C for the BEC^®^, and 93 °C for the bare copper surface. The solid-to-liquid thermal resistance decreased from 0.186 °C/W to 0.089 °C/W when boiling on the MuSEP coating compared to the bare copper surface. Also, the MuSEP coating exhibited the lowest thermal resistance at a lower power. The durability and effectiveness of the MuSEP coating were confirmed after passing more than 22,000 integrated hours of tests for functioning CPU in a two-phase thermosyphon cooling prototype and more than 5500 integrated hours in a total immersion cooling application. Figure 7 shows the scheme of the pool boiling setup and Figure 8 presents the schematic representation of the heater assembly.

**Figure 9:**
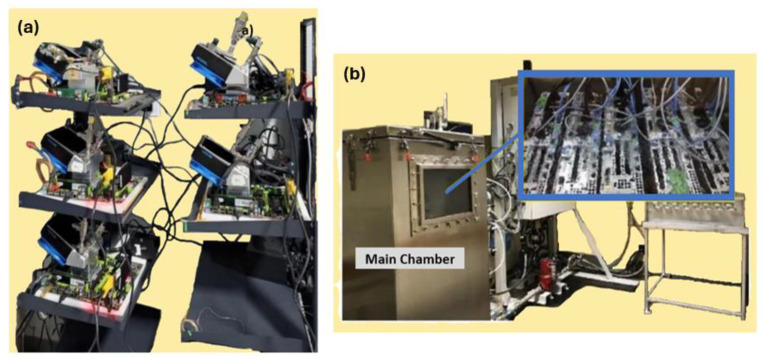
Reliability setups: (**a**) 4U heat sink system at 3IT; (**b**) total immersion system at Systemex Energies Incorporation. Adapted from [39].

The MuSEP coating was implemented in two systems for reliability tests presented in Figure 9.

### 2.4. Tunnels and Reentrant Cavities

Nakayama et al. [42] proposed a boiling improvement route consisting of initially gouging the boiling surface, presenting parallel rectangular channels with a width of 0.25 mm and a height of 0.4 mm; this was followed by covering the tunnels with a thin copper plate with rows of pores measuring 50 µm–150 µm. Employing such a methodology, the boiling curve for the R-11 refrigerant exposed tendencies mostly determined by the diameter and the density of the pores. The researchers suggested the following boiling modes using this enhancement surface: (i) The dried-up mode—here, the vapor phase fills the surface tunnels, and the boiling process is conducted outside the tunnels according to a mode similar to the one dictated by plain boiling surface with regularly spaced, active nucleation sites. (ii) The suction and evaporation mode—here, the departure of the bubbles from the active pores led to the fluid being sucked by the inactive pores into the tunnels; from here, the liquid spreads and evaporates, giving the best boiling heat transfer trend of all the boiling modes. (iii) The flooded mode—here, most of the surface tunnels are filled with the fluid and an active and isolated pore acta as the bubble nucleation site.

Moreover, Moita et al. [43] highlighted the relevance of bubble dynamics and interactions in the improvement of pool boiling; they stated that an inadequate regulation of these factors might lead to the generation of large bubbles, with noticeable coalescence and vapor blanket formation. In addition, Ji et al. [44] elaborated pool boiling for the R134a refrigerant outside tubes with reentrant cavities. Their results showed that the ideal heat-transfer-enhanced region seemed to be within 40 kW/m^2^ for the reentrant-cavity-enhanced tubes. Also, the heat-transfer ability was substantially increased at heat fluxes inferior to 200 kW/m^2^ and an increase in the HTC up to 330% was observed; this is superior to that of the plain heating tube. Nonetheless, at heat fluxes superior to 200 kW/m^2^, it was reported that the HTC of the enhanced tubes was inferior to that obtained with the plain heating tube.

Sun et al. [45] developed micro-grooved boiling surfaces, presenting reentrant cavities on copper substrates via the orthogonal ploughing–extrusion process and evaluated their pool boiling heat transfer trend using deionized water as the operating fluid. The experimental results confirmed that the micro-grooved surfaces with reentrant cavities exhibited a greater HTC than that achieved with a smooth copper substrate under all the different tested liquid subcooling conditions. The researchers noted that the micro-grooved surfaces with reentrant cavities suppressed the temperature excursion by reducing the wall’s superheat at onset nucleate boiling and did not reach the CHF in the experiment’s range. Additionally, the micro-grooved surfaces with reentrant cavities yielded a maximum HTC of nearly 38.8 kW/(m^2^·K) at 20 °C liquid subcooling, which was approximately 15.8% superior to the one obtained with a plain copper boiling surface. The authors also observed that there were two distinct types of bubbles on the boiling surfaces; these differed depending on whether they were generated in the reentrant cavities or in the microgrooves. The bubbles in the cavities were smaller than those on the microgrooves and remained practically unchanged with growing heat flux caused by the restriction associated with the diameter of the reentrant cavities. The diameters of the bubbles that were generated in the microgrooves were augmented at departure, with a rising heat flux of up to 465.6 kW/m^2^; then, after that, they decreased sharply for higher heat fluxes. Nonetheless, the imposed heat flux had only a minor impact on the departure diameter of the vapor bubbles nucleated in the surface reentrant cavities. With a growing liquid subcooling degree, the bubble diameter at the departure stage decreased, and the nucleate boiling HTC increased.

The work conducted by Pi et al. [46] was based on the development of reentrant microchannel structures via selective laser melting for pool boiling heat transfer amelioration. The 3D-printed reentrant microchannels were made of unique Ω-shaped reentrant microchannels with rough wall surfaces made of bronze powder. The authors performed pool boiling experiments of the reentrant microchannel structures using deionized water in saturated and liquid subcooled boiling scenarios with subcooling degrees of 15 °C and 30 °C, respectively. In view of the obtained experimental results, the researchers concluded that the reentrant microchannels aided in the boiling incipience, with lower wall superheats in respect to a smooth boiling surface. The rough wall surfaces of the reentrant microchannels structures presented many ripples and valleys, offering suitable sites for the vapor bubble nucleation to occur and triggering the onset of nucleate boiling in the much-reduced wall superheat. Moreover, the synthesized microchannel structures improved the boiling heat transfer by between 10% and 330% in comparison to the one obtained with a plain heating surface in subcooled boiling; the enhancements were more noticeable when small–moderate heat fluxes were imposed.

**Figure 10:**
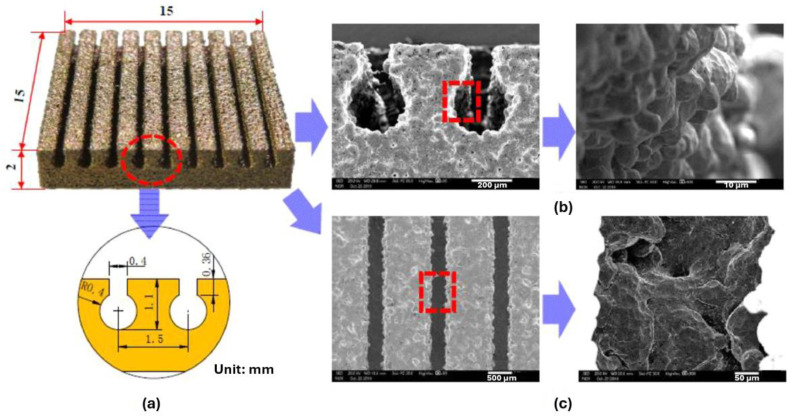
Three-dimensional printed reentrant microchannel structures and SEM imaging: (**a**) geometry of the reentrant microchannel structures; (**b**) SEM image of the reentrant microchannels cross-section; (**c**) SEM top view of the reentrant microchannel structures [46].

The researchers attributed the pool boiling heat transfer improvements to the pronounced enhancement of the density of the active nucleation sites and the extended heat transfer surface area, facilitating fluid replenishment for the heating surface rewetting trough the capillary wicking of the microscale channels. Nonetheless, the heat transfer increase was reduced at high-heat fluxes under saturated boiling conditions. The reentrant microchannels facilitated the separation of the flow pathways of the liquid–vapor counterflow and maintained the liquid replenishment and heating surface rewetting; thus, the system was enabled to obtain an ameliorated heat-transfer performance at heat fluxes up to 2900 kW/m^2^ in subcooled boiling. The heat transfer rate of the microchannels considerably increased in the subcooled boiling conditions in comparison to that of the saturated boiling conditions. The rising liquid subcooling degree promoted the enhancement of heat transfer at high and small heat fluxes; meanwhile, it had only a minor impact on the heat-transfer ability of the subcooled pool boiling of the reentrant microchannel structures at moderate heat fluxes. Figure 10 presents the geometric characteristics of the 3D-printed reentrant surface microchannels and corresponding SEM imaging.

Shete et al. [47] performed an experimental work to evaluate the nucleate boiling heat-transfer ability of reentrant cavity tubes used in R407C pool boiling at saturation temperature values of 7 °C and 10 °C and heat fluxes from 10.7 kW/m^2^ to 79.7 kW/m^2^. The boiling surface reentrant cavities provided a lower wall superheat than that achieved with a plain boiling surface. The reentrant cavities’ surfaces significantly augmented the density of active nucleation sites and facilitated the boiling nucleation at lower heat fluxes. The HTC increased with rising applied heat fluxes for the saturation temperature. The HTC of the developed surfaces using the R407c was between 1.3 and 1.6 times and between 1.4 and 1.8 times higher than that attained with a smooth heating surface at 7 °C and 10 °C, respectively, under equal heat flux. The experimental results indicated that the reentrant cavities with mouth size between 0.21 mm and 0.51 mm were promising candidates for use in increasing the boiling HTC of plain heating tubes. The 0.21 mm mouth showed an increased maximum HTC in comparison to the other tested mouth sizes. Also, the researchers proposed an empirical correlation for reentrant cavity boiling surfaces.

In general, surfaces with reentrant microstructures are among the most adequate structured boiling surfaces for pool boiling heat-transfer enhancement. The reentrant cavities or tunnels are widely recognized to act as vapor traps during the nucleate pool boiling process. Such vapor traps significantly aid the vapor bubble generation, increase the heat-transfer capability between the liquid and the solid surface, and delay the occurrence of the CHF. 

### 2.5. Wicking and Grooved Surfaces

Some published studies demonstrated that a monolayer wick of sintered copper particles heat transfer technique combined with wick tridimensional structures resulted in a high value for the HTC of 200 kW/m^2^·K and a CHF increased up to 6 times in comparison to that of a plain copper surface water pool boiling [48]. Nasersharifi et al. [27] synthesized many multilevel wicks of columnar, monolayer, and mushroom-shaped posts wicks for controlling the vapor and fluid flow for effective phase separation under pool boiling conditions using n-pentane as working fluid. The boiling surface was prepared with 200 µm copper particles by a multi-step sintering procedure. The monolayer wicks without and with the mushroom-shaped post structures provided 20% and 87% CHF enhancements, respectively, in comparison to a plain copper surface. Although the performance of the mushroom-shaped structure was like the one of the columnar posts’ wicks at low-heat fluxes, the mushroom-shaped wick considerably delayed the drying out of the heating surface as a result of a constant fluid supply through the mushroom-shaped cap. The enhancement in the CHF of the mushroom-shaped wick was attributed by the authors to the 3.5 mm spacing. A further spacing decrease to 1 mm resulted in a 250% CHF increase. The columnar and mushroom-shaped posts presenting a monolayer increased the HTC by a ten-fold in comparison to that of a plain boiling surface; this was because of the decreased conduction path through the thin monolayer wick in the vapor-regulated area employing the columnar and mushroom-shaped post wicks. It was observed that the liquid-filled monolayer appreciably reduced the superheat by decreasing the thermally conductive pathway; in contrast, the columnar posts adjusted the hydrodynamic instability wavelength to augment the CHF.

In the work presented by Raghupathi et al. [49], it was confirmed that extending the contact line regions within the base of a nucleating vapor bubble results in CHF enhancements. The formation of a fluid meniscus adjacent to the 10 µm–20 µm depth microscale grooves in the region of the base of the vapor bubbles was responsible for the extra contact line areas. The microgrooves’ depth was defined such that a sufficient reservoir of fluid was retained in the meniscus to maintain the evaporation process in the close-to-the-contact-line region throughout the vapor bubbles’ lifecycles. The pool boiling for water was conducted on copper surfaces with microgrooves with depths between 10 μm and 100 μm and widths between 100 μm and 500 μm pf; these were obtained by CNC machining. The authors found that, with shallow grooves measuring 10 μm in depth, the CHF was initially augmented by an increase in groove width, reaching a maximum of 1680 kW/m^2^ with a groove width of 300 μm; however, it decreased with further increases in groove width. The enhancement in the CHF was accompanied by an enhancement in the boiling HTC of up to 109 kW/(m^2^·K). The liquid evaporation was retained in the meniscus as the bubble grew over the microgroove walls; this was found to be the main contributor to the enhanced CHF instead of the wicking effect of the fluid. Moreover, for the 100 μm deep microgrooves, the CHF was augmented by the increasing width of the grooves, achieving a peak value of 1240 kW/m^2^; this was inferior to the one of a plain heating surface. The evolution of the CHF over the grooved boiling surfaces demonstrated that the distinct heat transfer mechanisms were affected by the depth of the surface grooves.

A study conducted by Deghani-Ashkezari and Salimpour [50] evaluated the impact of the geometry of the boiling surface grooves on the pool boiling process of water and an aqueous titania nanofluid. To enable the comparison of results, wire-cut machining was performed on a smooth surface of copper with several grooves having triangular, square, and semi-circular cross-sections and a pitch of 1.5 mm. The heat transfer regions of the plain boiling surface and the surfaces with square, semi-circular, and triangular grooves were approximately 1590.4 mm^2^, 3149.3 mm^2^, 2499.7 mm^2^, and 3542 mm^2^, respectively. The authors noted that the heating surface grooves enhanced the heat-transfer capability during the boiling process using water and nanofluids; in comparison to the performance of a plain boiling surface, the semi-circular and triangular grooved surfaces exhibited larger boiling HTCs. The square grooved surface exhibited the weakest thermal performance, and the HTC was enhanced only at low superheats. The triangular grooved heating surface presented the best pool boiling behavior among all the grooved surfaces and enhanced the HTC by up to 120%, 83.6%, and 65.6% when using only water and 0.2% vol. and 0.4% vol. concentrated nanofluids, respectively. The homologous values for the semi-circular grooved surface were 50.6%, 40.9%, and 34.7%, respectively. The enhanced heat transfer of the grooved boiling surfaces was caused by the augmented heat transfer area and the situation of the surface grooves. Moreover, the wicking and wetting effects of the surfaces had a considerable impact on the vapor film breakup and therefore on the boiling heat-transfer ability during quenching.

Li et al. [51] prepared wicking super-hydrophilic heating surfaces and hemi-wicking hydrophilic heating surfaces in the effort of exploring chemical etching techniques and nanoparticle deposition, respectively, onto spheres made of stainless steel. The saturated water quenching tests were conducted on such microporous boiling surfaces to evaluate the impact of the wickability effect of the surfaces on the vapor film’s collapse during the transition boiling regime. The wickability effect alters the liquid–solid contact mode in the transition boiling regime by enhancing the wetted area by capillarity. The extended area was determined through the synergistic action of the water flow’s instantaneous inertia impulse upon liquid–solid contact and surface tension. The researchers confirmed that the transitional heat flux at the critical transitional point that separated the transitional nucleate boiling and the transitional film boiling sub-regimes was appreciably increased with enhanced surface wickability. The best wicking surface led to a huge 656% maximum transitional heat flux enhancement in comparison to that achieved using a bare nonporous boiling surface. Also, the Weber number was changed to define the instantaneous water imbibition that occurred through the porous surface structures upon fluid–solid contact.

**Figure 11:**
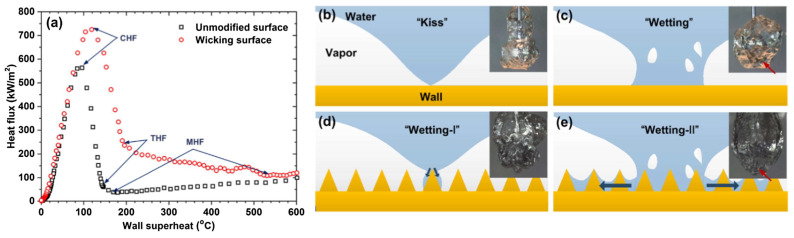
(**a**) Boiling curves of an unchanged boiling surface and on a wicking boiling surface, and schematic representation of the two sub-regimes during the transition boiling regime on the unchanged and wicking heating surfaces; (**b**) kiss mode or contact without wetting; (**c**) wetting mode; (**d**) wetting-I mode or kiss mode with wicking; (**e**) wetting-II mode or wetting mode with wicking [51].

The obtained results indicated that the transitional heat flux increase in ratio exhibited a linear correlation with the altered Weber number for the wicking and hemi-wicking surfaces; however, discrepancies were found between the slopes of the surfaces. This was most likely due to their distinct wettability. The authors also proposed a liner correlation between the enhancement ratio of THF and the modified Weber number, which was based on the hydrodynamic instability model. Figure 11 summarizes the main findings of the experimental work.

Serdyukov et al. [52] manufactured textured hemi-wicking silicon surfaces through laser ablation and evaluated the effect of the laser type on the silicon surface morphology and properties and water pool boiling heat-transfer enhancement. The authors performed surface texturing with lasers with visible (532 nm) and infrared (1064 nm) wavelengths, and with similar laser spot diameters and number of applied pulses per unit area.

The authors confirmed that the pool boiling behavior varied considerably according to the employed laser treatment: the infrared textured surface led to a 78% enhancement in the HTC when compared to the one obtained with an untreated silicon surface; meanwhile, the visible textured surface deteriorated the heat transfer rate from 5% to 40%, depending on the imposed heat flux. The same textured surface also exhibited a peak CHF value of 1806 kW/m^2^ that was more than two times larger than the one attained with the untreated silicon heating surface. This enhancement was attributed by the authors to the highest capillary wicking effect of the surface. The predicted values from the method introduced by the authors Rahman et al. [53] confirmed that, for infrared and visible laser-textured boiling surfaces, the wicking numbers were nearly 0.4 and 0.6, respectively.

In sum, numerical control machining, laser lithography, plasma technique, and wire electric discharge machining, among others, are commonly selected to produce pool boiling surfaces with structures holding channels and grooves. These structures enhance the surface roughness, increase the effective heat transfer surface area, increase the number of active nucleation sites, enhance wicking ability, and form separate pathways to aid the nucleation and growth of the bubbles and their detachment from the surface. As a result, the boiling performances are significantly improved. However, the high costs that are involved and the complexity of fabrication somewhat hinder the large-scale application of these surfaces. Nonetheless, the properties of these structured surfaces indicate their improved thermal performance at pool boiling scenarios. Particularly, the grooved surfaces have been proven to be effective in enhancing boiling heat transfer, given that the grooves provide nucleation sites and act as conduits for liquid flooding caused by bubble lifting and/or capillary wicking, improving both phase-change heat transfer and convection heat transfer. Also, more studies on the impact of the dimensions of the grooves on the pool boiling heat-transfer behavior and HTC are most welcome. This parameter directly influences the nucleation of bubbles, liquid–vapor separation, and liquid entrance length. Excessive nucleation activity will generate too many bubbles that could block the fluid pathway, whereas too much space for the liquid pathway will reduce the nucleation of the bubbles too much.

### 2.6. Tuned Wettability Surfaces

The already published studies revealed that hydrophilic and hydrophobic surfaces have a considerable capability for nucleate pool boiling heat-transfer enhancement. Advances regarding the alteration of the surface energy of the heating surface have created opportunities for improving the pool boiling heat transfer by altering the surface contact angle with the inclusion of nanostructures forming a porous layer on the surface during the boiling process. Such a layer alters the wettability of the surface and improves the CHF and boiling performance. All the involved mechanisms prove that the modification of surface wettability is a methodology that is capable of effectively increasing the heat-transfer characteristics in pool boiling scenarios.

Betz et al. [54] verified that hydrophilic surfaces delay film formation and the film boiling regime, whilst a hydrophobic surface augments the population of active nucleation sites. The authors examined different surface patterns, including hydrophilic and hydrophobic surfaces, for pool boiling enhancement and reported improved values of HTC and CHF for all the tested surface patterns: maximum enhancements of 65% were achieved for the boiling HTC and maximum enhancements of 100% were achieved for the CHF as compared to a bare surface. In addition, Takata et al. [55] produced super-hydrophobic surfaces by coating with nanoparticles of nickel and polytetrafluorethylene that achieved a water contact angle of 152°. The production of a vapor film on the heating surface was observed before the initiation of the nucleate boiling regime. The bubbles surged at the saturation point and coalesced with each other to form a film of vapor. However, it is noteworthy that the hydrophobic coating enabled improved heat transfer.

Phan et al. [56] used metal–organic chemical vapor deposition, plasma-enhanced chemical vapor deposition, and nanoparticle deposition during the process of nanofluid pool boiling to prepare distinct wettability surfaces; this enabled an investigation of the influence of the contact angle on the water boiling heat transfer. The obtained hydrophobic surfaces lowered the incipience superheat but hindered the release of bubbles and caused the spreading and coalescence of the bubbles at high-heat fluxes, resulting in the production of a vapor blanket over the entire heating surface. The produced surface, with its hydrophilic nature, provoked an enhancement in the bubble departure diameter, but the bubble departure frequency was reduced with increasing wettability. Also, a surface with contact angles ranging from 45 to 90° exhibited a decrement in the HTC with reducing contact angle. Nevertheless, the surfaces presenting contact angles inferior to 45° presented an enhancement in the nucleate boiling heat-transfer performance, reducing the contact angle. The highest HTC was achieved employing a heating surface with a contact angle approaching zero.

Furthermore, Moze et al. [57] prepared laser-textured aluminum boiling surfaces employing a nanosecond fiber laser and the deposition of a fluorinated silane by chemical vapor deposition to alter their wettability from super-hydrophilic to super-hydrophobic. The larger microcavities (4.2 μm of average diameter) induced an early transition into the favorable nucleate boiling regime, whereas the smaller microcavities (2.8 μm of average diameter) offered improved thermal behavior at large heat fluxes. Consequently, both super-hydrophobic and super-hydrophilic surfaces showed appreciable enhancements in the pool boiling heat-transfer capability; the super-hydrophobic surface achieved a considerable HTC enhancement of 500%.

Patankar [58] found that the hydrophobic nature had the merit of the earlier boiling incipience, but also had the limitation of a smaller CHF, whereas the hydrophilic character enhanced the nucleate boiling heat transfer at the expense of a delayed boiling incipience. Hence, the authors developed an enhanced nucleating heat transfer surface with hydrophobic pores atop a hydrophilic substrate. Also, Jo et al. [59] explored a hydrophobic Teflon coating and a hydrophilic silica coating on silicon surfaces to evaluate the influence of their wettability on the pool boiling heat-transfer behavior. The hydrophobic boiling surfaces were observed to ameliorate the nucleate boiling heat transfer rate using water at small heat flux values; the hydrophilic surfaces exhibited better performances at high-heat fluxes. The authors also concluded that biphilic surfaces [60] composed of hydrophilic base region and a profusion of hydrophobic dots led to a better boiling heat-transfer performance than either the homogeneous hydrophobic or hydrophilic boiling surfaces. Nevertheless, the CHF for the developed heterogeneous heating surface was similar to the one obtained when using the homogeneous hydrophilic surface.

In addition, Betz et al. [61] examined biphilic, super-biphilic, super-hydrophilic, super-hydrophobic, hydrophilic, and hydrophobic boiling surfaces. The super-hydrophilic surfaces had a near-zero contact angle and the super-hydrophobic ones exhibited a water contact angle higher than 150°. The biphilic surfaces were composed of hydrophilic and hydrophobic surfaces; the super-biphilic surfaces were produced by juxtaposing super-hydrophilic and super-hydrophobic surfaces. It should be emphasized that the improved HTC and CHF values were achieved using biphilic surfaces in comparison to those obtained with homogeneous-wettability boiling surfaces. The enhanced values of more than 100 W/cm^2^ for the CHF and more than 100 kW/(m^2^·K) for the HTC were determined with the super-biphilic heating surfaces.

Bertossi et al. [62] developed a switchable wettability boiling surface employing thermo-responsive polymers coated on a stainless-steel surface. When the temperature of the heating surface reached the switching point of the polymer, which is slightly higher than the temperature of the working fluid’s saturation, the wettability of the polymer altered from hydrophilic to hydrophobic; therefore, it promoted vapor bubble nucleation. During the bubble growth, the heat conducted near the triple-phase contact line provoked a localized decrease in the polymer temperature, which switched the wettability back from hydrophobic to hydrophilic. Nevertheless, the HTC enhancement for water was relatively limited, with a result of around 20%.

Additionally, a study conducted by the authors [63] presented an enhancement of boiling heat transfer on super-hydrophilic, hydrophobic, and micro-patterned hydrophobic/super-hydrophilic surfaces. With this purpose, stainless-steel foils were air-sprayed with a coating composed of silica and PDMS with high hydrophobicity due to its hierarchical structure and the use of a hydrophobic polymer. The coating was treated with a pulsed Nd/YAG laser to produce biphilic patterns. The homogeneous super-hydrophilic surface had the highest CHF value, which was more than 350% of that achieved on the uncoated stainless steel. The authors observed that the increased wettability reduced the bubble contact diameter, allowed a higher density of active nucleation sites, and delayed the drying out of the surface. An opposite trend was observed on the hydrophobic surface, where the vapor film covered the surface after boiling incipience. However, this surface provided a high HTC at reduced heat fluxes, since the first bubble appeared on the surface at a superheat of less than 1 K. The biphilic surfaces with hydrophobic spots of 2 × 2 mm^2^, 1 × 1 mm^2^, and 250 × 250 μm^2^ area exhibited an increase of up to 200% in CHF as compared to the uncoated stainless steel. The highest HTC of 51.2 kW/m^2^ was achieved on the surface with the smallest hydrophobic spots, given that those reduced the bubble diameter and increased the nucleation frequency. The surfaces with larger hydrophobic spots promoted the boiling incipience and exhibited higher HTC values at low-heat fluxes. With increasing heat flux, the vapor film started to cover the hydrophobic regions, reducing the heat transfer.

Furthermore, Pontes et al. [64,65] studied the heat transfer during single-bubble nucleation on biphilic surfaces composed of hydrophilic regions surrounding super-hydrophobic regions. The authors observed that the bubble dynamics were influenced by the dimension of the super-hydrophobic regions. Since bubbles are constrained to the boundaries of the super-hydrophobic regions that change the diameter of the base of the bubbles, the smaller super-hydrophobic regions tend to promote the stable generation of vapor bubbles due to the greater surface tension on the regions near the hydrophilic–super-hydrophobic boundaries nearby. With an increase in the base diameter, the surface tension, which only acts at the boundary with the hydrophilic region, decreases. Consequently, the smaller super-hydrophobic regions promoted a greater evaporation mass transfer rate. The temperature gradients were higher at the boundaries and induced convection of the cold fluid in the super-hydrophobic regions’ pitch.

Teodori et al. [66] studied the heat transfer and bubble dynamics in water pool boiling using surfaces exhibiting super-hydrophobicity and hydrophilicity. The wettability was adjusted by modifying the surface chemistry without fluctuations in the surface roughness. The authors observed a specific trend for the boiling curve of the super-hydrophobic surfaces, as the heat flux increased almost linearly with growing superheat in the wall; however, here, there was a lower slope than that seen for the hydrophilic surfaces. This was derived from the formation of a stable vapor blanket over the surface at only 1 K of wall superheat due to the immediate coalescence of the bubbles. This behavior agrees with the results of the quasi-Leidenfrost regime and with the results of heat flux theoretical prediction proposed by the authors. The results confirmed that the existing models could estimate the general tendencies of the bubble growth, employing its contact angle in the case of the hydrophilic surfaces. Approximating the modified contact angle with the quasi-static contact angle that was obtained during surface characterization was useful for a qualitative evaluation; however, the results did not support its use when predicting the bubbles’ departure diameters. It was concluded that, in the case of super-hydrophobic surfaces, the effect of the vapor film should be considered.

Yu et al. [67] numerically investigated the pool boiling process on biphilic surfaces using a three-dimensional thermal multiphase lattice Boltzmann model with liquid–vapor phase change. The surface was textured with square pillars composed of hydrophilic sides and hydrophobic tops. The simulations were performed to infer the impact of the wettability of the hydrophilic regions and the pillar width and height. It was reported that an appropriate increase in the contact angle of the hydrophilic region promoted the bubble nucleation on the substrate and, hence, enhanced the nucleate boiling on the biphilic surface, but a larger contact angle of the hydrophilic region could make the boiling enter the film boiling regime. The heat flux initially increased with growing pillar width and then exhibited a declining trend after reaching its maximum. The optimal contact angle of the hydrophilic region and pillar width diminished with the growing wall superheat, confirming that the hydrophilic region of the mixed-wettability surface played an increasingly leading role in the rising of the wall’s superheat. The orthogonal array tests were performed, and it was verified that the wettability of the hydrophilic regions was the dominant influencing factor.

**Figure 12:**
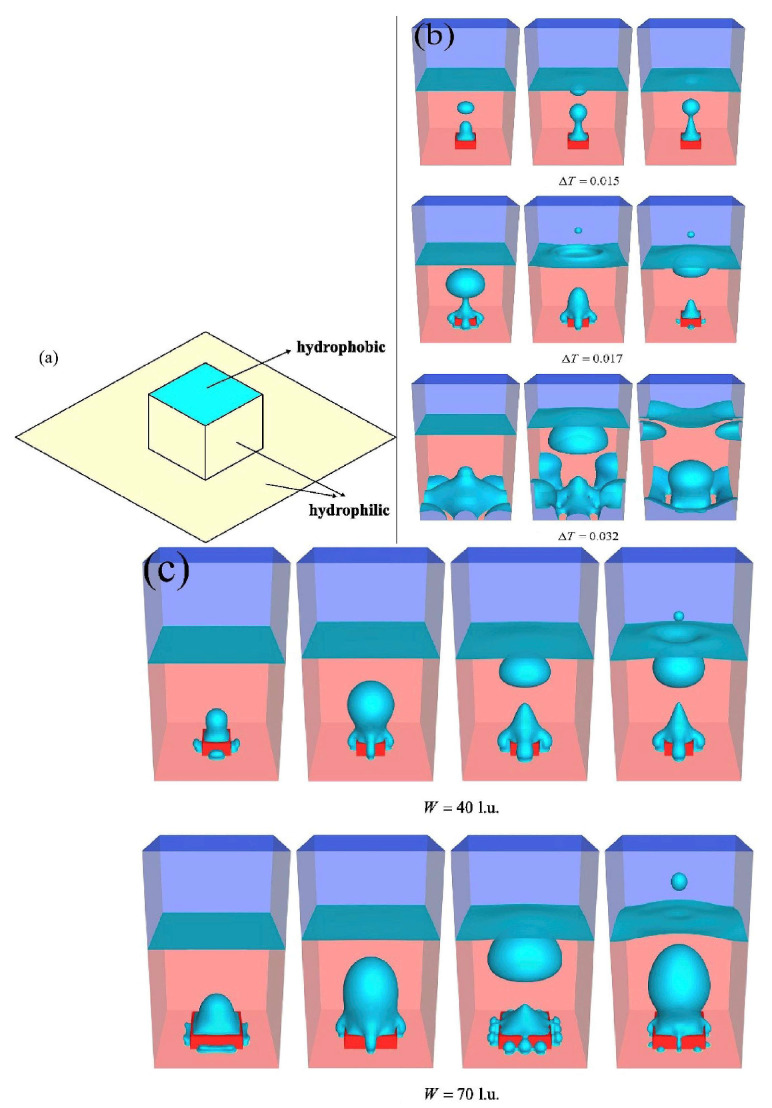
Snapshots of the pool boiling process at different superheats: (**a**) ΔT = 0.015 °C, (**b**) 0.017 °C, and (**c**) 0.032 °C. From left to right: t = 7000 δ, 12,000 δ, and 18,000 δ. Contact angles θphi ≈ 45.3° and θpho ≈ 103°. The pillar width and pillar height are taken as w = 40 l.u. and w = 20 l.u., respectively. δ represents the time step and l.u. represents lattice units [67].

Figure 12 shows the boiling snapshots on the hydrophilic–hydrophobic mixed surface at different wall superheats of 0.015, 0.017, and 0.032. It can be seen from the figure that, in the case of a wall’s superheat that is equal to 0.015, the bubbles are just nucleated on the top surface of the pillar or hydrophobic region of the mixed-wettability surface, and there are no bubbles nucleating on the bottom substrate. With the enhancement of the wall superheat from 0.015 to 0.017, some bubbles were also nucleated on the bottom substrate, as seen in Figure 12b, which enhanced the nucleate boiling heat-transfer ability. When the wall superheat was increased to 0.032, a large portion of the heating surface was covered by a vapor blanket and the boiling process entered in the transition boiling regime, as shown in Figure 12c.

Additionally, Shim et al. [68] synthesized patterned super-biphilic surfaces having distinct super-hydrophobic area fractions and performed pool boiling heat transfer experiments with them as the boiling surfaces. The authors observed that the developed super-biphilic surfaces with wicking effect presented simultaneous enhancements of the CHF and HTC. The local super-hydrophobic dots facilitated the nucleation of vapor bubbles and stabilized the density of active nucleation sites to cause large increases in the boiling HTC. The super-hydrophilic region, which were made of silicon nanowires, possessed a super-hydrophilic character and wicking action to enhance the CHF through a fast fluid supply to the dry regions. To determine the CHF and HTC, the pool boiling experiments were performed using homogeneous wettability and super-biphilic boiling surfaces.

**Figure 13:**
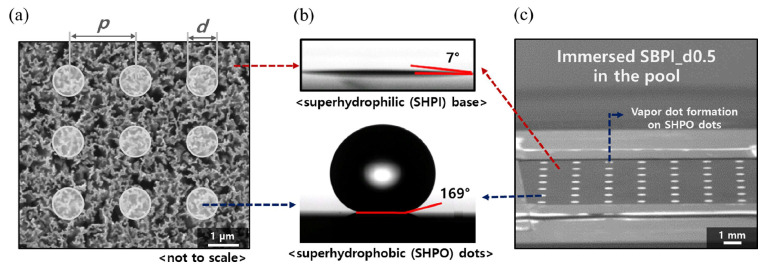
Super-biphilic surfaces with silicon nanowires and coating with PFOTS: (**a**) SEM image of the silicon nanowires, and parameters of the super-hydrophobic regions of the super-biphilic surfaces; (**b**) contact angle of super-hydrophilic regions and super-hydrophobic dot regions; (**c**) immersed images of SBPI_d0.5 in a pool of deionized water [68].

The obtained results showed that the super-biphilic surfaces augmented the boiling HTC; this was the case regardless of the super-hydrophobic fraction of the surface, because of the promoted nucleation and the maximized number of active nucleation sites. The CHF was additionally enhanced on the SBPI_d0.1 surface compared to the H-SHPI surface; meanwhile, in other cases, the CHF decreased. This result overcomes the pending issue of CHF reductions on the super-biphilic surface with wicking due to the suppression of liquid supply to the super-hydrophobic regions. The authors attributed the CHF enhancement to the reduction in the super-hydrophobic area of the surfaces, which maintained the super-hydrophilic and wicking surface area for rapid microscale fluid supply to the dry regions of the surface. Also, another contributor was the fact that the macroscale fluid supply was ensured by the separation of the vapor and liquid pathways to prevent the transition into the film boiling regime. Also, the researchers proposed a dimensionless liquid supply factor, which was employed to confirm the relationship with the CHF. Figure 13 illustrates the fabrication process of the developed super-biphilic surfaces.

Specifically, biphilic or super-biphilic patterned surfaces have been widely utilized to enhance HTC and CHF. However, it remains a challenging issue to improve CHF on super-biphilic surfaces with wicking phenomena due to the suppression of the liquid supply in the hydrophobic regions. In the present work, to investigate the mechanism and experimentally break through the limits of CHF enhancement, artificially patterned super-biphilic (SBPI) surfaces with different super-hydrophobic (SHPO) area fractions were produced; pool boiling experiments were conducted. By artificially promoting nucleation, all SBPI surfaces demonstrated a higher HTC than the homogeneous wettability surfaces did. Considering dynamic wicking and bubble behaviors, the SBPI successfully broke through the CHF of the homogeneous super-hydrophilic surfaces. It is concluded that the non-dimensional liquid supply factor, which reflects both wicking and bubble behaviors, is essential to design structured surfaces during boiling. The results can contribute to a strategy for further improving boiling performance by controlling wettability on nanoscale interfaces.

Overall, the studies on biphilic surfaces demonstrated that hydrophilic surfaces with hydrophobic dots were effective in improving the heat-transfer performance. The super-biphilic and biphilic heating surfaces were demonstrated to be very suitable options; this is because the size and number of the hydrophobic regions defined the density of the nucleation sites and the average diameter of the vapor bubbles. However, one conclusion that can be made relates to the scarcity of available sufficiently sized databases for the concepts related to biphilic surfaces. Therefore, the longevity of the wettability-adjusted surfaces in harsh pool boiling scenarios remains a serious challenge; further studies are required concerning the cost-effectiveness and durability of the pool boiling heat-transfer performance using biphilic surfaces, so that a better understanding can be formed. Particularly, the biphilic or super-biphilic patterned heating surfaces have been widely used to increase the CHF and HTC. Nonetheless, it remains a challenge to enhance the CHF on super-biphilic surfaces with wicking phenomena because of the suppression of liquid supply in the super-hydrophobic regions. Consequently, further studies are needed to investigate the underlying mechanisms and to break through the limits of CHF enhancement with artificially patterned super-biphilic surfaces.

### 2.7. Surface Roughening

Surface roughness has a profound impact on heat-transfer performance, including single-phase and two-phase convection and radiation [69]. Increasing the roughness of the heating surface by techniques like sandblasting, chemical etching, mechanical roughening, and induction of artificial cavities is a commonly followed route for ameliorating the nucleate’s boiling performance. The reason behind such enhancement is to attain a higher number of nucleation sites on the heating surface.

Also, Anderson and Mudawar [70] reported on a remarkable improvement in the nucleate boiling heat transfer for the FC-72 commercial coolant; one sanded heating surface had parallel gouges measuring from 0.6 µm to 1.0 µm, and one vapor-blasted porous heating surface with pore sizes measuring near 15 µm. The authors attributed such enhancement to the greater population of active nucleation sites.

Furthermore, Kim et al. [71] confirmed the profound dependence of water CHF on the surface roughness. The CHF was found to increase from 775 kW/m^2^ for a smooth surface with a R_a_ of 0.041 µm to 1625 kW/m^2^ for a rough surface, having an R_a_ of 2.36 µm. The research team ascribed this enhancement to the improved capillary wicking effect on the rougher surface. In addition, the authors proposed Equation (1), accounting for the wicking effects:

(1) $$q_{CHF}^{\prime \prime }=0.811\left(\frac{1+cos\alpha }{16}\right)\cdot {\left[\frac{2}{\pi }+\frac{\pi }{4}\left(1+cos\alpha \right)+\frac{351.2cos\alpha }{1+cos\alpha }\left(\frac{R_{a}}{S_{m}}\right)\right]}^{1/2}\cdot \rho_{g}h_{fg}{\left[\frac{\sigma g\left(\rho_{f}-\rho_{g}\right)}{\rho_{g}^{2}}\right]}^{1/4}$$

where α is the static contact angle and S_m_ is the mean spacing between the surface peaks. Moreover, though several published works have already demonstrated the benefits of the surface roughening enhancement procedure, this form remains not commonly adopted in large-scale applications. The major factor behind the absence of the commercial use of roughened enhanced surfaces is the quick aging effect of the surface features, usually lasting only for a few hours, during which the pool boiling heat-transfer performance gradually deteriorates back to the one of a plain boiling surface.

One demonstration of this was the study performed by Chaudhri and McDougall [72], in which the authors evaluated the long-term boiling heat-transfer behavior using heating surfaces with parallel scratches measuring 0.025 mm in width. The researchers also demonstrated that the enhancement for perchloroethylene and isopropyl acetate was merely temporary, and the thermal performance after the boiling process for a few hundreds of hours abated back to that of a plain bare surface. Hence, it is required that further studies on the surface roughening enhancement involve an accurate assessment of the aging effects. The surface roughness can be characterized through the mean roughness R_a_ or effective heat transfer area ratio r (the ratio of the actual heat transfer area and its projected area).

One example is the study by Moita et al. [43] who verified that surfaces with larger r could not assure a larger boiling HTC. The authors showed that a higher r could enhance the liquid–solid contact area, improving the HTC; however, at the same time, it may cause the early formation of a vapor blanket due to the coalescence of the bubbles. This would degrade the HTC and even overcome the benefits of the increased liquid–solid contact area. Also, several researchers found an enhanced number of nucleation sites on heating surfaces with greater R_a_, which was believed to be responsible for the boiling HTC augmentation. However, some different observations were also reported, like the ones stated by Benjamin and Balakrishnan [73]. They observed that the number of nucleation sites did not consistently increase with R_a_: it decreased at first and, after that, increased with growing R_a_.

The main difficulty in fully understanding the influence of the heat transfer surface roughness on pool boiling arises from the inconsistency of experimental data, which results from different roughening techniques, measurement uncertainties, and different conditions, such as the working fluid, surface material, and wettability. Particularly, the need of using a consistent boiling surface roughening technique when studying its impact on the pool boiling heat-transfer ability was investigated by [74], given that heat transfer surfaces produced by certain roughening methodologies have a higher tendency toward the occurrence of bubble nucleation, regardless of the surface roughness. In addition, the surface roughness parameters R_a_ and R_q_ are statistically obtained values which are unable to accurately translate the real texture of the surface, especially concerning the number of nucleation cavities. Hence, a smooth heating surface with small R_a_ or R_q_ can have a considerable number of cavities that are suitable for nucleation.

Some authors like Luke [75] have already proposed methodologies to measure the surface-active cavity size to quantitatively determine the potential nucleation sites. However, such approaches follow the simplistic treatment of the surface texture and wettability. Hence, the quantitative prediction of the nucleation sites remains very difficult or even unrealizable, and authors must characterize the surface roughness with statistic roughness parameters.

Moreover Fan et al. [76] prepared eight testing surfaces on plain copper via random polishing, unidirectional polishing, and femtosecond laser machining, attaining average surface roughness ranging between 0.045 µm and 1.35 µm. The surface roughness was characterized by the areal three-dimensional roughness parameter. The water pool boiling process showed that a rougher boiling surface had higher HTC than a smoother surface, only within the same surface preparation method; this was the case if the surface roughness was characterized by *Sa_p_*. For the surfaces prepared through diverse techniques, the laser-processed rough surface with *Sa_p_* equal to 3.4 µm could have deteriorated the boiling HTC compared with the relatively smooth one polished by the 180-grit sandpaper with an *Sa_p_* equal to 1.3 µm. Nonetheless, a distinct tendency was verified in the cases where the surface roughness was defined by the *Sa* parameter. Upon the previous application of a filtering process, the large-scale component of the surface can be eliminated; the areal arithmetical mean height can be determined from the remaining part, denoted as *Sa*. The boiling curves shifted to the left with rising *Sa*, independently of the surface preparation methodologies.

The enhancement in the HTC of rougher surfaces was the result of a greater number of active nucleation sites, the reduced diameter of the detached bubbles, and a greater bubble departure frequency. Also, the CHF did not exhibit a clear evolution with the surface roughness when the different surface preparation methods were used, regardless of whether *Sa_p_* or *Sa* was employed. The contact angle measurements indicated that the CHF was not only dependent on the surface roughness, but also closely linked to the boiling surface wettability.

The different surface roughening techniques could introduce different surface characteristics and wettability characters. The unidirectionally polished surface possessed many irregular scratches, whereas the femtosecond laser-processed surface showed fluctuating surface texture. For the sandpaper-polished surfaces, the contact angle initially increased with growing surface roughness and remained unchanged as the surface roughness augmented beyond a threshold limit. The tendency was the result of the sessile droplet spreading onto the surfaces. A rougher surface with greater scratches could impede the droplet from spreading, particularly in the direction normal to the scratches. A rougher surface could exhibit an increased contact angle since the spreading of the droplets is hindered. This tendency was interrupted for a surface roughness *Sa* superior to 0.6 µm; beyond this, the obstruction effect of the droplet spreading encountered a threshold value.

Further increases in the surface roughness would not enhance the hydrophobic character of the heating surface. In addition, the contact angles of the heating surfaces prepared through the femtosecond laser were of approximately 153°, and the roughness of the surfaces had only a minor effect on the contact angle. Through a standardized filtration process of the surfaces, their boiling curves shifted to the left with rising surface roughness parameter *Sa*, independently of the surface synthesis methodology. The surface-roughening method had only a minor impact on the HTC once the large-scale surface texture—surface form and waviness—were properly removed from the surface. In addition, the CHF was influenced by the surface roughness and the surface-roughening method. It was not a monotonic function of surface roughness without describing the surface-roughening method.

The wettability of the surface also played a relevant role in determining the CHF. The rough surfaces enhanced the boiling heat-transfer ability by providing more active nucleation sites, reducing the diameter of the vapor bubble at the departure stage, and increasing the bubble’s departure frequency. Additionally, the population of nucleating vapor bubbles augmented with increasing heat flux, which did well to explain that the boiling HTC can be defined by an increasing function of heat flux in all boiling curves. Moreover, the research team developed a correlation to predict the HTC from the experiments as a function of *Sa*, especially in the fully developed process of nucleate boiling.

In essence, it can be stated that the relationship between the HTC and the boiling surface roughness is somewhat clarified. However, the influence of the surface roughness on the CHF remains unclear and requires further in-depth study. Several researchers argue that the CHF can be triggered by the hydrodynamic instability effect by this way, excluding the surface roughness and the wettability effects. Also, there remains the discussion of whether the surface roughness or surface wettability are the dominant influencing factors of the CHF enhancement in the pool boiling process. A major part of the experimental works related to the surface roughness’s impact on the CHF demonstrated varying enhancements in the CHF with growing surface roughness. Although surface roughness shows somewhat limited enhancement of CHF for metal boiling surfaces, some studies on the influence of the surface roughness on hydrophilic silicon surfaces confirmed greater increases in the CHF in comparison to the metal heating surfaces. Moreover, some authors have focused on the combined effect of the surface structure and wettability on silicon surfaces. 

### 2.8. Extended Surface

The production of finned heating surfaces with rectangular or square fins is a commonly used procedure to achieve boiling heat-transfer enhancement. The primary benefit of such a methodology is the increased surface area for heat transfer; the main challenge is the optimization of the dimensions of the fins and the pitch between them to reach an optimal thermal performance.

Wei et al. [77] found that the CHF for FC-72 and boiling surfaces with square micro-fins increased with the growing height of the fins for a fixed fin width; in contrast, for a fixed non-dimensional roughness value, they found that the CHF value was augmented by increases in the fin width. The authors attributed the boiling heat-transfer enhancement to the evaporation and micro-convection effects of the superheated fluid in the pitch between fins. The research team also found that the HTC was not affected by the orientation of the boiling surface, and the CHF for the vertical heating surface was 20% lower than that of the horizontal surface.

Moreover, Chu et al. [78] observed that, with water as the thermal fluid, the CHF for finned surfaces was augmented by increased non-dimensional roughness from 1.79 to 5.94. The authors modified the model introduced by Kandlikar [79] to better account with the surface tension from the bubbles, and proposed Equation (2) for the CHF from finned surfaces:

(2) $$q_{CHF}^{\prime \prime }=\frac{1+cos\alpha }{16}{\left[\frac{2}{\pi }\left(\frac{1+\alpha_{m}}{1+cos\alpha }\right)+\frac{\pi }{4}\left(1+cos\alpha \right)cos\theta \right]}^{1/2}\cdot \rho_{g}h_{fg}{\left[\sigma g\left(\rho_{f}-\rho_{g}\right)/\rho_{g}^{2}\right]}^{1/4}$$

where r^+^ is the non-dimensional roughness, α is the contact angle of the fluid on a plain heating surface, α_rec_ is the receding contact angle of the fluid on a plain heating surface, and α_m_ is the modified contact angle of the fluid on a finned surface. This can be given by Equation (3):

(3) $$\alpha_{m}=r^{+}cos\alpha_{rec}$$

In addition, McNeil et al. [80] carried out pool boiling experiments using R-113 and water as operating fluids, and a heat sink made of copper with pin fins with a cross-section of 1 × 1 mm^2^, a height of 1 mm, and a spacing of 2 mm, arranged in a line. The goal of the work was to infer the impact of the substrate’s thickness and the effectiveness of the fins on the boiling performance. Also, it should be noted that the effective heat transfer area ratio corresponds to the geometric heat transfer area ratio in cases where the efficiency of the fins is equal to one and, in this case, the geometric ratio was 1.75.

The finned area of the boiling surface had lower impact on the pool boiling performance with a decreasing intrinsic thermal conductivity of the surface. Consequently, a high heating surface thermal conductivity was needed for the extended surface, but a uniform heat flux cannot be attained with a high surface thermal conductivity. The peak in the heat flux at the heat sink inlet diminished with the decrease in the surface thermal conductivity. However, the extended boiling surface was rendered ineffective. The influence of the thickness of the substrate was also assessed by altering its total thickness whilst maintaining one half of the thickness as copper and the other half as aluminum. With decreasing surface thickness, the effective heat transfer area ratio was maintained, and the heat flux became more uniform. Nonetheless, even at a thickness of 1 mm, a heat flux that could be considered as uniform was not achieved, having noticeable distortions in the inlet and outlet nearby regions of the heat sink. Nevertheless, further studies are required to provide guidelines about the optimal fin size, geometry, pitch, and configuration for different types of thermal fluids, operating conditions, substrate orientations, thicknesses, and thermal conductivities.

Additionally, Kong et al. [81] carried out an experimental work to examine the pool boiling heat-transfer behavior employing innovative bi-structured heating surfaces with a micro-pin-fin structure. The micro-pin-finned and plain regions were distributed on the boiling surface via the dry etching process. The tested substrates were silicon chips with the following surface morphology: smooth silicon chip; a surface of PF30-60 with micro-pin-fins measuring 30 μm at the side with a height of 60 μm; a surface of PF30-60LS comprising extended plain strips in a micro-pin-finned region; a surface of PF30-60SS, with short strips in a micro-pin-finned region; a surface of PF30-60LP, with extended plain passages in a micro-pin-finned region; a surface of PF30-60SP, with short passages in a micro-pin-finned region. The FC-72 refrigerant was used as the operating fluid.

The results showed that the bi-structured surfaces effectively enhanced the heat-transfer ability in the nucleate boiling process, with the CHF also being appreciably enhanced. It could be observed that the bi-structured surfaces led to an increased number of vapor bubbles, given that a great number of active nucleation sites were generated in the micro-pin-finned regions; the small bubbles grew, collided, merged, and moved quickly to the adjacent plain channel. When the vapor bubbles grew large enough, they detached rapidly under the force of the channel pressure. Moreover, the micronization phenomenon also showed that the boundaries between the enhanced structured regions and the plain ones were active nucleate site lines.

The CHF enhancement of bi-structured surfaces was more than 120% and 15% in comparison with the CHFs of a smooth silicon chip and a PF30-60 surface, respectively. Furthermore, the wall superheat at onset nucleate boiling of the PF30-60SP/SS heating surface was reduced; at the same heat flux, the temperatures of the PF30-60SP and PF30-60SS surfaces were decreased by around 10 K and 8 K, respectively, in comparison to that of the PF30-60 surface. Despite the bi-structured boiling surfaces with smaller surface area enhancement ratio, the micro-pin-finned region provided active nucleation site density greater than that of the PF30-60t surface. The developed bi-structured heating surfaces optimized the bubble dynamics and enhanced the mass and heat transfer, releasing the limitation associated with the thermal resistance. The authors also stated that the width of the channels impacted on the vapor bubbles’ departure diameter. In the case of a grown bubble presenting a diameter like the width of the strip, the pressure in the surface channels provided an extra upward force to facilitate the detachment of it from the boiling surface. Consequently, the smaller channeled surfaces exhibited a better fluid supply and lower wall superheat when compared to those achieved with the larger-channeled surfaces.

In essence, producing heating surfaces presenting multiple rectangular/square fins is one of the most popular approaches for improving the performance of the pool boiling heat transfer process. The obvious benefit of such a type of surface is the increase in the heat transfer area; the main challenge lies in finding an optimal fin width and height and an ideal spacing between them (pitch) for attaining an overall optimal heat-transfer performance.

### 2.9. Displaced Enhancement

The heat-transfer enhancement technique known by displaced enhancement involves the use of metallic foams [82,83] and metallic mesh attachments, static mixers, screen lamination, and the use of different kinds of objects fitted over the heating surface, like plates, rings, disks, manifolds, and objects and structures of complex geometry. An improved fluid mixing, effective liquid–vapor pathway separation, and pressure recovery are some of the merits of this heat-transfer enhancement technique.

Tsay et al. [84] studied the impact derived from covering a heat transfer surface using a metal mesh and enhancing the surface roughness on the water pool boiling process. The mesh enhanced the heat-transfer performance, particularly for a shallow-water-layer-type approach, when the measurements of the mesh were similar to the diameter of the bubbles when they detached from the surface. Nonetheless, in the case where it was applied over a rough heating surface, the metal mesh compromised the effectiveness of the heat transfer. The exploration of a metallic foam with a porosity degree superior to 90% has attracted considerable interest from researchers because of its low weight and heat-transfer enhancement capability.

For instance, Xu et al. [85] evaluated the acetone pool boiling process using a copper foam for enhancement purposes. The researchers observed that the main influencing thermal performance factor was the number of pores per inch. A foam with a reduced number of pores per inch exhibited improved heat transfer at reduced the liquid wall superheat; in contrast, a foam presenting with a higher number of pores per inch provided a better performance for moderate–large superheating processes. In addition, the thermal performance and the nucleation site density were augmented by higher foam layer thicknesses, but with a thicker foam, the resistance to vapor release also increased.

Yang et al. [86] investigated the pool boiling behavior using a copper foam welded onto a copper heating surface. The authors reported that the heat transfer substrate decreased the incipience superheat, increased the CHF by up to three times, and appreciably reduced the onset of the water nucleate pool boiling in comparison with a smooth copper boiling surface. The authors obtained a maximum heat-transfer enhancement, employing a foam with 60 pores per inch, and concluded that the boiling performance depended on both the number of pores per inch and the thickness of the copper foam.

In a study performed by Hayes et al. [87], multiple small hollow conical structures were printed above an aluminum boiling surface to promote liquid–vapor separation. The authors considered the hypothesis of using fluid inertia and buoyancy forces above the heating surface to develop structured fluid and vapor flows. The vapor and fluid flows above the heat transfer surface were adjusted by incorporating hollow conical structures produced by metal additive manufacturing onto the heating surface. The sides and top of the hollow conical structures had holes to enable the vapor bubbles generated under them to be removed, whilst also allowing the bulk liquid to rewet the heating surface. As the operating fluid boiled and the vapor phase was trapped inside the structures, separated fluid and vapor flow pathways were generated at their holes. The effective vapor removal and short fluid flow extension over the heating surface provided enhancements in the heat-transfer capability. Hence, the boiling performance improvement offered by these structures was caused by the modulation of the fluid flow field and the secondary boiling outside the hollow conical structures was caused by an increased population of nucleation sites.

**Figure 14:**
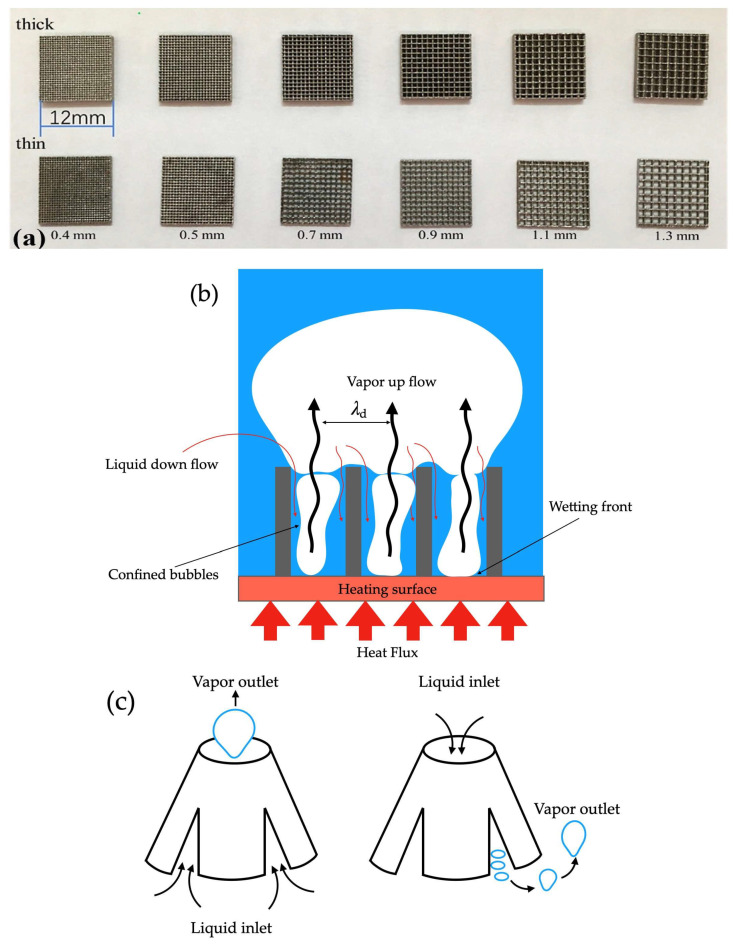

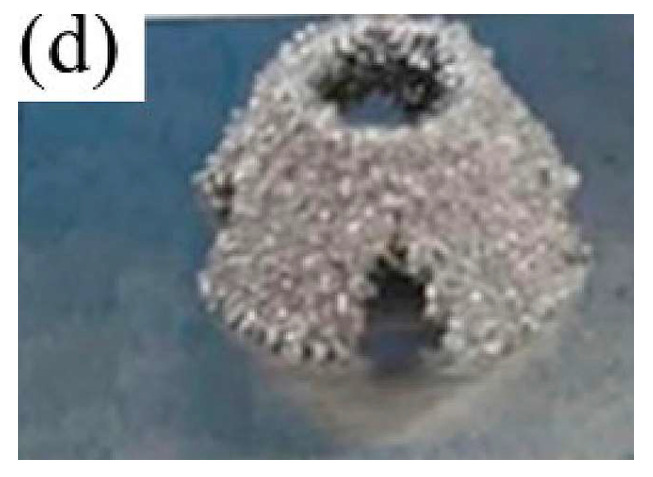
Images and schematic representation of the developed hollow conical structures via 3D printing for the separation of the vapor and fluid flow pathways: (**a**) parts of the conical hollow structures with different thicknesses; (**b**) liquid and vapor flows inside the hollow conical structure; (**c**) schematic illustration of the separation of the liquid and vapor pathways; (**d**) hollow conical structure over the aluminum boiling surface [87].

The results revealed that 74% of the boiling enhancement attained with this approach was derived from convective heat transfer action. The boiling performance was further increased using the same structures over microchannels to ameliorate the heat transfer in the entrance region. With the combination of multiple miniaturized hollow conical structures with microchannels, an HTC of 190 kW/m^2^·K was obtained; this represents a 4-fold increase in comparison to the HTC of a smooth aluminum heating surface. Also, this study was the initial step for demonstrating the ability of the additive manufacturing to produce surface structures exhibiting macro-convection to ameliorate the boiling heat-transfer behavior. Figure 14 presents images and schemes of the developed hollow conical structures.

The dual tapered microgap concept was extended to pool boiling experiments in the work conducted by [88]; here, a CHF of 2880 kW/m^2^ was obtained using a plain copper surface and water as the operating fluid. Increases of 2.3-fold in the HTC and CHF were achieved employing a dual tapered arrangement. The vapor bubbles squeezing- and pressure-recovery mechanisms within the tapered microgap created a self-sustaining fluid-pumping action, which promoted a two-phase stable flow and continuous surface rewetting at large heat fluxes; this way, the heat-dissipation capability of the system was enhanced.

Chauhan and Kandlikar [89] evaluated the pool boiling heat-transfer performance using a dual tapered microgap and a dielectric operating fluid. The objectives were to infer the impact of the taper angle and the height of the inlet on the HTC and CHF, and to estimate the HTC through a proposed model. In this sense, taper angles from 5° to 25° and inlet gap heights of between 0.8 mm and 1.3 mm were employed with the HFE-7000 commercial refrigerant. A reduction in the CHF was observed for the 5°, 10°, and 15° taper angles, with two mentioned inlet gap heights because of the incoming effect from the lower-pressure recovery using smaller taper angles. Also, an increased boiling HTC was achieved for all the taper angle options in the comparison, compared to a configuration without a manifold block.

The HTCs obtained with taper angles of 20° and 25° and an inlet gap height of 0.8 mm were 13.5 kW/(m^2^·K) and 17.8 kW/(m^2^·K), respectively, when a CHF value of 300 kW/m^2^ was reached. In the case of an inlet gap height of around 1.3 mm, the reached boiling HTC for 20° and 25° taper angles was of 14.5 kW/(m^2^·K). Enhancements of 2-fold and 1.5-fold in the HTC were reported with the 25° taper angle and inlet gap heights of 0.8 mm and 1.27 mm, respectively, as compared to the configuration without a manifold block. The model derived from the homogeneous fluid flow model and boiling heat transfer correlation was explored to estimate the HTCs of different configurations.

For the cases where the pressure recovery balanced the pressure drop with sustained fluid flow, the boiling HTC was estimated and validated through comparison with experimental data. The peak deviation between the predicted and experimental results of the HTC was of around 13.3% using the 15° taper and an inlet gap height of 0.8 mm. The results indicated a deteriorated heat-dissipation capability when employing smaller taper angles. The model showed poor pressure recovery in the case of small taper angles and, thus, unstable vapor bubble expansion was expected to happen in the microgap conducting to decreases in the CHF.

When dealing with higher taper angles, the pressure recovery balanced the pressure drop because of the geometry, momentum change, and friction. In these cases, very high values of HTC were achieved, and the model predicted accurately the heat-transfer characteristics. The pressure recovery process together with the tapered microgap length and vapor bubbles squeezing were the main aspects that were found to be responsible for the enhanced heat dissipation ability of the tapered configuration. The mentioned mechanisms provided stable liquid flow and pumping effect in the microgap. The tapered gap microchannels provided an enhanced structure, modifying the fluid flow field above the heating surface by producing a unidirectional flow generated by the forces of the vapor bubbles growth stage.

Khalaf-Allah et al. [90] conducted pool boiling tests with the combined usage of nanoparticles added to water and a rotating mixer above the heating surface as a pool boiling enhancement technique. The authors employed a circular brass plate as a boiling surface and used varying concentrations of alumina nanoparticles. The rotation velocity of the mixer varied between 350 rpm and 800 rpm. The obtained results indicated a 58.8% increase in the boiling HTC at an imposed heat flux of 280 kW/m^2^ and 0.03% vol. concentration of alumina nanoparticles suspended in water.

**Figure 15:**
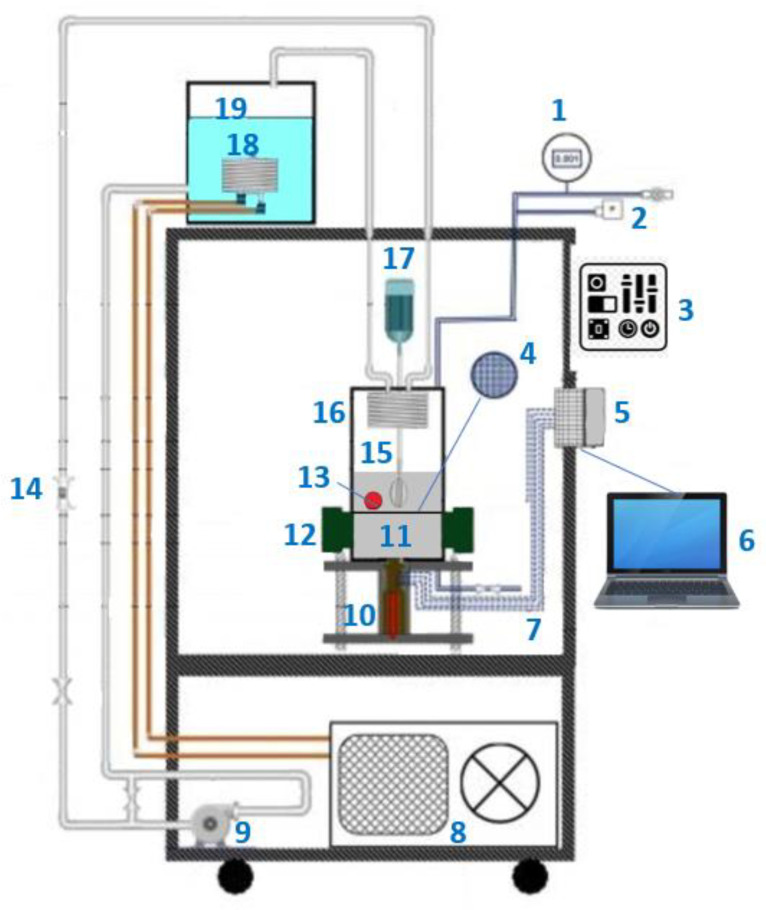
Pool boiling setup: 1—pressure gauge; 2—pressure cutout; 3—control panel; 4—wire mesh layer; 5—data acquisition; 6—laptop; 7—K-type thermocouples; 8—condensation unit; 9—water pump; 10—heating block; 11—boiling surface; 12—side-view glass; 13—pre-heater; 14—flowmeter; 15—paddle mixer; 16—condenser coil; 17—mixer motor; 18—chilled evaporator; 19—water tank. Adapted from [90].

The research team found that the HTC was enhanced by up to 23.6% when using only the alumina aqueous nanofluid, while it attained a 27.1% enhancement in the HTC in the cases where only the rotating mixer was employed under similar working conditions. Figure 15 displays the scheme of the pool boiling setup.

The authors also verified that the HTC was augmented by the rising concentration of the nanoparticles, up to the mentioned percentage of 0.03% vol., but it decreased for higher alumina concentrations. The HTC enhancement increased with the increasing rotation speed of the mechanical mixer. The used compound enhancement technique prevented the sedimentation of the alumina nanoparticles on the heating surface in comparison to what occurred when using the alumina nanofluid. Also, it was stated that the hybrid technique of the incorporation of nanoparticles of alumina to stirred water above the boiling surface enhanced the HTC by 40% in comparison to the one obtained with an iron oxide aqueous nanofluid and the applications of an external magnetic field.

Overall, it can be stated that almost all the published papers on pool boiling using metal foams report a considerable heat-transfer enhancement in comparison with the plain/flat boiling surfaces. The foams promoted a higher CHF and HTC and a decreased wall superheat at the onset of the nucleate boiling. The fundamental beneficial features of the foams are the increased specific surface area, the capability for disturbing strong fluid, and wetted area enhancement; these facilitate the generation of vapor bubbles at low-heat fluxes together with an increased density of active nucleation sites at high-heat fluxes. The main parameters impacting on the pool boiling heat transfer by metal foams are the density of the pores or pores-per-inch, porosity (ratio between the void volume and the total volume of the foam), and the thickness of the foams. The wetted area increases with growing density of pores of the foam. An increased pore density will make the pore diameter decrease. The small pores augment the capillary-driven liquid flow towards the foam. Nonetheless, the small pores prevent the bubble departure at high-heat fluxes. Also, thicker foams exhibit an enhanced wetted area and an increase in the HTC in low-heat fluxes; in contrast, the same thicker foams degrade the nucleate boiling heat-transfer performance at high-heat fluxes because of the thermal resistance produced by the vapor trapped in the porous structure. However, the effect of the foam thickness requires further experimental works to clarify the underlying mechanism of enhancement.

Regarding the displaced enhancement of the pool boiling heat transfer, if the pool boiling is assisted by a mechanical agitator, the heat transfer is dominated by forced convection and the heat transfer from the surface increases. It is known that the choice of agitator in vessels with agitation is made according to the density of the fluid. The mechanical agitators rotating at low velocities improve the heat-transfer ability when using a high-viscosity operating fluid; meanwhile, the agitators rotating at high velocities promote the heat transfer when using low-viscosity working fluids. Taking a blade agitator as example, it should be positioned above the heating surface to enhance the combined effect derived from the buoyancy force, the drag force, and the turbulence intensity. Nevertheless, it is highly recommended that more studies are conducted on the influence of the number of blades, the rotational velocity, and the distance between the agitator and the boiling surface on the pool boiling HTC.

### 2.10. Bi-Conductive Surfaces

The use of bi-conductive surfaces is promising in pool boiling heat-transfer enhancement. The advantageous features of the bi-conductive surfaces in the pool boiling amelioration have already been reported; however, the detailed enhancement underlying mechanism has not yet been totally understood because of the lack of information on factors like velocity, temperature, and the two-phase interface trend.

In the same research scope, Yim et al. [91] elaborated pool boiling tests employing copper and bi-conductive boiling surfaces, deionized water, and a sodium dodecyl sulfate surfactant. The research team argued that the onset of the nucleate boiling of the surfactant solution happened earlier than that of the deionized water, and the boiling heat-transfer ability of the system was improved under medium- and low-heat fluxes. Nevertheless, the surfactant solution exhibited a smaller CHF. Through a vapor bubble behavior analysis, the research team observed that the mushroom-shaped bubbles and the small bubbles coexisted and affected each other during the process of surfactant solution pool boiling. Also, the small bubbles break up throughout the mushroom-shaped bubbles and accelerate the growth and further detachment of the mushroom-shaped bubbles, decelerating the rising of the temperature of the surface after the boiling crisis.

**Figure 16:**
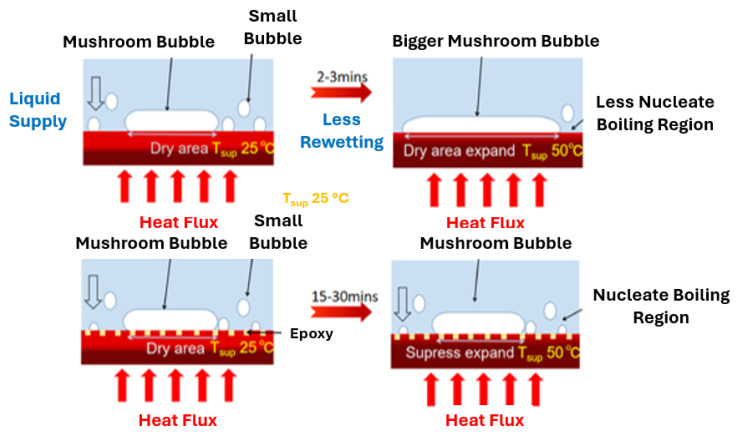
Comparison of the heat-transfer characteristics between the copper surface and the bi-conductive surface using a schematic diagram of the temperature and bubble dynamics on the two boiling surfaces [91].

The nucleate boiling heat-transfer capability of the bi-conductive surface was like that of the copper heating surfaces in the early stage of nucleate boiling. With further increases in the applied heat flux, the heat-transfer performance offered by the two boiling surfaces becomes divergent. The increase rate of the temperature of the bi-conductive surface was slower than the one reported for the copper surface, and it exhibited a larger CHF. The researchers also confirmed that the material with poor thermal conductivity hindered the lateral heat transfer, and ensured the nucleation of smaller vapor bubbles, inhibiting the expansion of the drying out of the surface regions. Figure 16 schematically represents the bubble dynamics and the temperature of the two heating surfaces.

Deng et al. [92] analyzed the phase change in the pool boiling heat-transfer behavior using bi-conductive heating surfaces composed by a highly thermally conductive substrate with embedded low-thermal-conductivity insets. In their work, the nucleation, growth, and detachment from the bi-conductive surface bubble dynamics stages were reproduced and the temperature and fluid flow trends during pool boiling were assessed.

In view of the experimental results, the authors concluded the following: (i) The underlying mechanism of the boiling improvement brought by the bi-conductive surfaces lay in the pinning of the bubble contact lines. Limiting the contacts of the bubbles prevented the coalescence of the lateral vapor bubbles and ensured the strict vertical growth of the bubbles. Since the contact region of the restricted bubbles and the surface was limited, the bubbles’ departure frequency raised considerably; therefore, the HTC and CHF on the bi-conductive surfaces were enhanced as well. (ii) The boiling process was maximized when the embedding space of the inserts was similar to the operating fluid capillary length. Also, a too-small space led to bubble coalescence before their growth and a too-large space led to multiple-bubble nucleation and coalescence. Such behaviors could result in the formation of a local vapor film, which in turn may reduce the boiling performance. (iii) Enhancing the width of the insets improved the HTC under low superheats, but it also led to CHF enhancement; meanwhile, changing the depth of the insets had only a negligible impact on the boiling heat-transfer performance on the bi-conductive surface. (iv) The bi-conductive boiling surfaces led to an increase in the CHF through embedding a poor-thermal-conductivity material into the bulk substrate with high thermal conductivity; hence, it showed a reliable ability for pool boiling heat transfer improvement.

Moreover, Heidary et al. [93] addressed the impact of low-conductive channels on the deionized water pool boiling heat-transfer capability. The authors prepared copper heating surfaces with channels presenting distinct depth and width values by the wire electric discharge machining. Regarding the surface channels, three different modes were studied: empty channels, alternately filled channels, and filled channels. These were studied using an epoxy–silica aerogel mixture as a hydrophilic low-conduction substance.

**Figure 17:**
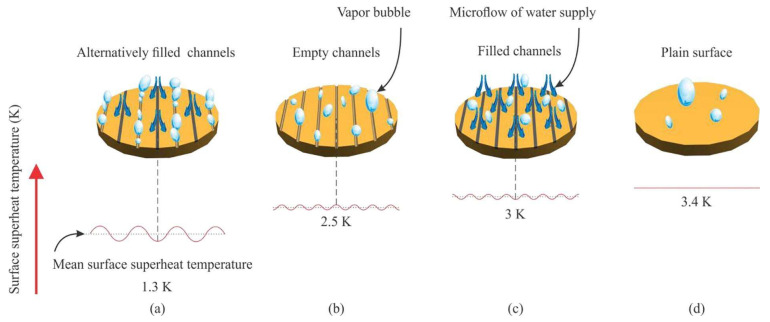
Schematic representation of the onset of nucleate boiling in the different boiling surfaces: (**a**) surface with alternatively filled channels; (**b**) surface with empty channels; (**c**) surface with filled channels; (**d**) plain surface. The figure includes the location of the active nucleation sites, the effect of low-conductive channels on supplying water to the surface, and the surface temperature distribution [93].

The authors concluded that the utilization of wide, hydrophilic, low-conductive channels increased the contact angle of the entire sample surface from 1.5° to 9.8°, which depended on the position of the water droplet. At considerable heat fluxes, if the channels’ depth was not augmented, then the best heat-transfer behavior was obtained with surfaces presenting filled, alternatively filled, and empty channels. For surfaces with alternatively filled channels, an increase in the boiling of HTC up to 174% could be achieved at small heat fluxes in comparison to the one obtained reported for a plain heating surface. In such boiling surfaces, the generation of vapor bubbles happened in the empty channels, and the filled channels produced microscale flows. The enhancement of the boiling heat transfer surface area provoked by the empty surface channels exhibited a smaller impact on the pool boiling process amelioration than the dimensional characteristics of the channels. A comprehensive empirical correlation was proposed to estimate the heat flux of bi-conductive boiling surfaces, presenting diverse geometrical dimensions of the channels with a mean absolute error of around 9%. Figure 17 shows a schematic representation of the onset of nucleate boiling in the different boiling surfaces.

Shakeri et al. [94] investigated the impact of changing by response surface methodology; at the same time, they examined the influencing factors of the electrodeposition process, proposing an intensified route promoter of the pool boiling process through the preparation of hybrid bi-conductive boiling surfaces. The duration, current density, and number of electrodeposition steps were changed to evaluate the thickness, porosity, wickability, and boiling HTC of the coated surfaces. The pool boiling tests were carried out employing deionized water under atmospheric conditions. The authors found that, as the number of electrodeposition steps was increased, the dependence of the wickability action on the current density and duration period diminished, given that the number of electrodeposition steps did not considerably impact the morphology.

Isolating the coated surface from a 10-step electrodeposited surface and shining light through it led to a surge of noticeable white dots that could be the vapor-phase flow pathways from the surface base during boiling. Increasing the deposition steps decreased the porosity degree because of the filling of the surface cavities with the prolonged deposition period. With a deposition period shorter than 32 s, it was not feasible to create a porous structure or to enhance that period by more than a 44 s conduction to a roughened surface morphology. The electrodeposited porous layer thickness was built up by enhancing the number of steps. A surface was manufactured with a current density of 600 mA/cm^2^, a deposition period of 50 s, and an eight-step layering process; this surface led to the best pool boiling enhancement, with an electrodeposited surface that enhanced the peak heat flux and boiling HTCs by approximately 46.8% and 238.9% in comparison to a plain heating surface, respectively.

**Figure 18:**
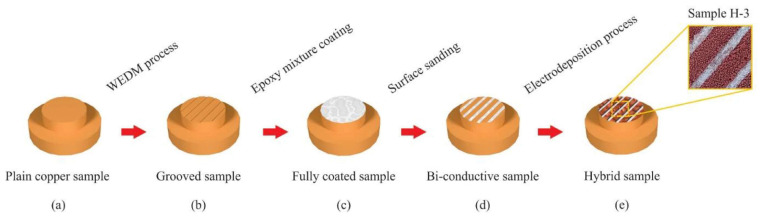
Main sample preparation steps: (**a**) plain copper sample after CNC cutting; (**b**) production of channels; (**c**) coated sample using a mixture of epoxy glue and silica aerogel particles; (**d**) bi-conductive sample prepared through manual sanding; (**e**) final bi-conductive sample prepared through electrodeposition [94].

The proposed experimental correlations were taken for the influencing factors such as porosity, thickness, wickability, and HTC through the regression models. The boiling surface, having electrodeposition layers that were 2.5 mm wide and poorly conductive channels which were 1.5 mm wide in between, possessed the maximum HTC and heat flux of around 177 kW/(m^2^·K) and 1192 kW/m^2^, respectively. Figure 18 illustrates the main steps of the preparation of the samples.

In sum, the use of low-thermal-conductivity materials embedded on the boiling surface created fluctuations in the surface temperature. These materials can be explored for the preparation of bi-conductive boiling surfaces composed of two different regions. A higher-temperature region presents active nucleation sites for vapor bubble generation; a constantly wet region promotes the supply of operating liquid to the boiling surface. The bi-conductive heating surfaces are favorable for pool boiling heat-transfer enhancement, especially when submitted to high-heat fluxes. Apart from this benefit, the bi-conductive surfaces usually exhibit a greater structural strength, durability, reduced thermal performance deterioration over time, and involve no susceptible structures or coatings. Though the advantageous features of the bi-conductive boiling surfaces are well-established, a detailed description of the underlying mechanisms of heat-transfer enhancement remains out of reach; this is due the absence of data related to temperature, velocity, and two-phase interface evolution. Hence, further studies on this subject are welcome.

## 3. Surfactants and Polymers

The pool boiling performance of the common heat-transfer fluids including water, ethylene glycol, and coolants is determined by their promising thermophysical characteristics, but also by the low values of specific properties that limits their effectiveness, particularly at high-heat fluxes. With the inclusion of additives into the fluid, it is reasonable to adjust properties like surface tension, leading to improvements in the nucleate pool boiling behavior. One of those additives is surfactant, which is an organic amphiphilic compound containing long-chain molecules with hydrophilic heads and hydrophobic tails.

In this area of research, Wen et al. [95] used quaternary ammonium cationic surfactant solutions of decyl trimethylammonium bromide (DETAB), dodecyl trimethylammonium bromide (DOTAB), myristyl methylammonium bromide (MTAB), and cetyltrimethylammonium bromide (CTAB) at various concentrations in their pool boiling experiments; they aimed to examine the pool boiling performance of their aqueous solutions using a copper heating surface with and without applying an external electric field. The obtained results revealed that the aqueous surfactant solution could substantially increase the boiling HTC in comparison with the one obtained with distilled water only.

The boiling HTC exhibited increments with rising concentration until a critical micelle concentration and, after that, HTC levels off. The comparative peak HTC enhancement can reach up to approximately 209%, with this maximum being attributed to the enhanced number of active nucleation sites and the increased vapor bubble departure frequency. In addition to the decrease in the surface tension, the repulsion force from the electrostatic interaction is also responsible for the changes in the density of the nucleation sites and the bubble departure frequency. Nonetheless, the CHF showed a continuous decrement with the increasing concentration of the surfactant. When employing a surfactant solution with low concentration, the CHF is even slightly superior to the one of pure water. This is due to a surface wettability improvement after the boiling process. The CHF will be inferior with the use of moderate and high surfactant concentrations. The surfactants exposing a lower critical micelle concentration tend to exhibit greater HTCs and lower CHFs at low concentration values. Nevertheless, in the case of a comparatively high concentration, in respect to the critical micelle concentration, the four types of quaternary ammonium cationic surfactant solutions are prone to present the same boiling curves.

The authors concluded that incorporating a surfactant into water will slightly augment the viscosity, which shows short increments with growing concentration. While the surfactant solution has a lower surface tension than that of distilled water, the decrease in the surface tension augments with growing concentration until the critical micelle concentration and, after that, levels off. The HTC of the surfactant solution was considerably higher than that of the water, with an improvement of more than 2 times for the peak boiling HTC. Such an enhancement was comprehensively ascribed to the decrease in the surface tension and the surfactant-induced electrostatic repulsive force. These two factors together lead to a denser nucleate site, a smaller bubble departure diameter, and a faster departure frequency in surfactant solution boiling; together, these factors form the explanation for the higher derived HTC.

The CHF decreased continuously with the increasing concentration of the surfactant. The CHF using low-concentration surfactant solutions were even slightly superior to the one obtained with water, presenting comparative enhancements from 15% to 24%. With the concentration rising, the CHFs of the surfactant solutions started to become lower than those obtained using water, with a maximum decrease of 23.7% in the 3200 ppm solution. The CHF increment in the low-concentration solution was attributed by the authors to an improved surface wettability after surfactant solution boiling. In high-concentration solutions, the effect of surfactant overwhelms the surface wettability. Although the surface still has a super-hydrophilic character, an earlier occurrence of film boiling happens due to the presence of surfactant, which subsequently leads to a smaller CHF.

Overall, the surfactant with a smaller CMC tends to have a higher maximum HTC and a lower CHF under a low concentration. However, the situation changes with further rising of the surfactant concentration. For a relatively high concentration compared to CMC, all the surfactant solutions tend to have a similar boiling curve, with an approximate CHF and a maximum HTC. Also, comparative experiments with different electric field implementations were carried out to infer the influence of the electric field on the boiling characteristics.

In view of the results, the electric field was found to impose only a negligible effect on the pool boiling performance when using the surfactants. New bubble dynamic phenomena named bubble entrainment and liquid entrainment were observed during visual observations. Many small bubbles that were generated at the heating surface were absorbed into a big bubble, leading to the phenomenon called bubble entrainment, with the departure of large bubbles from the heating surface. Similarly, “liquid column in a big bubble” (called as “liquid entrainment”) is observed in surfactant boiling tests.

Guo et al. [96] conducted pool boiling tests using ultra-pure water and a surfactant aqueous solution of Triton X-114. The boiling surface was made of transparent bare fluorine doped tin oxide. Different applied heat fluxes and subcooling degrees of 5 °C, 25 °C, and 35 °C were used. It was confirmed that, with the reduction in the liquid subcooling, an obvious vapor bubble coalescence occurred when using ultra-pure water as the operating fluid. Meanwhile, with increments in the liquid subcooling degrees, the vapor bubbles’ surfaces became unstable and rough. Also, the newly discovered mechanism of the bubble–bubble penetration happened in the surfactant solution under similar operating conditions as those with the ultra-pure water. The bubble–bubble penetration could be defined as a bubble interaction in the absence of any coalescence. Such vapor bubble behavior was ascribed to the micro-convection caused by the bubble base shrinkage and the Marangoni effect from the gradient of the surface tension. The combined action of the Marangoni effect and the motion of the bubble surface provoked an aggregation of the molecules of the surfactant toward the base of the bubbles, facilitating the nucleation of the next vapor bubble. Owing to the rapid growth stage of the newly generated bubbles, the bubble–bubble penetration and piercing phenomena occurred during the growing of the bubbles. The freshly generated vapor bubble partially accelerates the initial bubble detachment from the surface, also interacting with the first bubble by forming a tiny vapor channel. This phenomenon should be one of the main reasons for the improved heat transfer using surfactant solutions.

Through pool boiling tests using ultra-pure water and surfactant solutions, distinct types of vapor bubble behavior were observed. The bubble–bubble penetration phenomenon was revealed in the nonionic surfactant solution. The influence of the different heat fluxes and subcooling degrees on the pool boiling heat transfer were studied. The authors concluded that the behavior of the bubbles in surfactant solutions differed from those with ultra-pure water. The existence and movement of the surfactant changes the surface tension and its distribution, leading to the phenomenon of the bubble–bubble penetration, piercing, and double penetration, among other phenomena, with these phenomena being more noticeable at small heat fluxes.

In contrast, when using the ultra-pure water, it was mainly determined through bubble coalescence. The adsorption of surfactant on the solid–liquid interface aids in the quick nucleation of the next vapor bubble. The redistribution of the concentration of surfactants in the solution and heating surface during the growth and detachment of the bubbles can be considered the fundamental underlying mechanism for the vapor bubble–bubble penetration. With rising heat flux, the bubble behavior in the surfactant solution becomes more active, from the previous bubble–bubble penetration, the bubble emission, to the quick coalescence. Also, with the rising of the liquid subcooling, the surface became more unstable and rougher; this was caused by the intense condensation on the front of the vapor–liquid interface.

Levitskiy et al. [97] proposed that, with a low polymer concentration of 0.01 wt. %, the generation of normal stresses deforming the liquid layer between a growing bubble and the heating surface increased the bubble departure and, consequently, increased the HTC. However, a high polymer concentration (e.g., 1 wt. %) degraded the boiling performance as the enhanced fluid viscosity resisted the bubble growth and suppressed the micro-convection near the surface. An overall conclusion can be deduced: the extent of the pool boiling enhancement with surfactants and polymers depends on the concentration, type, and chemical composition, as well as the heat flux. But, given the contradictory findings from different studies, these mechanisms warrant further profound investigation. Aside from the dependence of any boiling enhancement on the additive type and concentration, there exists a critical concentration for optimum enhancement, which complicates the additive selection and work with proper concentration.

The authors concluded that the addition of a surfactant into the fluid shifts the nucleate boiling region towards lower surface superheats, thereby provoking an earlier boiling incipience and an increase in the boiling HTC. The maximum boiling performance enhancement was achieved at an optimal surfactant concentration, and exceeding this concentration degraded the performance because of the increase in the fluid viscosity. The boiling enhancement was caused by surface tension, surfactant adsorption and desorption, greater number of nucleation sites, foaming, alterations in bubble dynamics, and improved surface wettability. The effects of the addition of a polymer into the thermal fluid on the boiling heat transfer are polymer-dependent. The polymers may improve the HTC below the optimum concentration, or degrade the thermal behavior, depending on the changes in the fluid viscosity provoked by their addition. The enhancement is caused by reduced bubble coalescence and enhanced nucleation, and the deterioration is attributed to the reduction in the bubble growth rate, the reduction in the contact angle, and the suppression of the micro-convection.

Luo et al. [98] employed polyvinylpyrrolidone, having various polymerization degrees for improving the nucleate pool boiling heat transfer at mass fractions ranging between 100 ppm and 3200 ppm. For PVP K15, K60, and K90, their highest heat transfer coefficients were 141.9 kW/(m^2^·K) at 3200 ppm, 150.6 kW/(m^2^·K) at 3200 ppm, and 141.5 kW/(m^2^·K) at 1600 ppm, respectively; these are all much higher than the 61.4 kW/(m^2^·K) of distilled water. Alterations in the active nucleation sites, bubble size, and detachment from the surface frequency were verified with the observation of specific behaviors including bubble departure retardancy, bubble repulsion, bubble–bubble penetration phenomenon, and fluid entrainment. A higher polymerization degree corresponds to higher localized viscosity, lower surface tension, larger normal stress between the vapor bubbles and the heating surface, and higher repulsion force between the vapor bubbles.

In comparison to the traditional surfactants in terms of pool boiling enhancement, the polymers possess better safety properties; meanwhile, the impact of the polymerization degree is a lack of investigation. Also, the PVP K90 solution presented the smallest surface tension and the sharpest viscosity enhancement. Concerning the bubble dynamics, changes in the number of nucleation sites, bubble dimensions, and detachment frequency were confirmed, with the detection of several special behaviors including bubble repulsion, bubble leaving retardancy, bubble–bubble penetration, and fluid entrainment.

**Figure 19:**
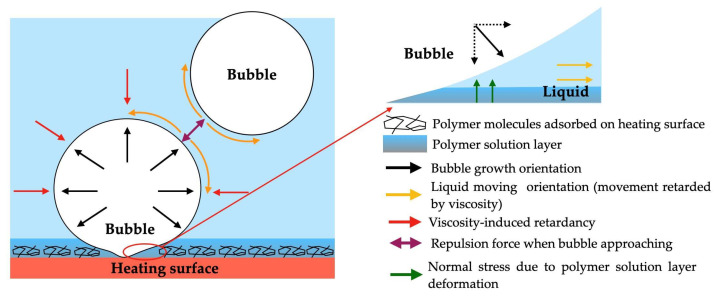
Schematic representation of the nucleate pool boiling process in polymer solution.

The effect of the polymerization degree was comprehensively deduced at a concentration lower than the critical concentration of the polymer. In essence, the polymerization degree impacts the adsorption nature on surface and the local solution properties near the bubble boundary. Higher polymerization degrees correspond to lower surface tension reduction, higher local viscosity, stronger normal stress between bubble and surface, and greater repulsion force between bubbles. The combined effect leads to changes in the boiling HTC. Incorporating low-concentration polymers with elevated polymerization levels was expected to be beneficial for the improvement of the nucleate boiling behavior. Figure 19 displays a schematic drawing of the nucleate pool boiling in polymer solution.

In essence, it can be stated that adding a surfactant into the operating fluid will shift the nucleate boiling region toward lower surface superheat degrees, thereby promoting an earlier boiling incipience and enhancing the pool boiling HTC. The maximum thermal performance enhancement is attained for an optimal concentration of surfactant that increases with the decreasing molecular weight of surfactant; exceeding the ideal concentration may reduce the enhancement effect due to dynamic viscosity increase in the operating fluid. The underlying mechanisms that are usually highlighted as being responsible for the heat-transfer enhancement are the surfactant adsorption and desorption at the liquid–vapor interface, the surface tension, the Marangoni convection, the enhanced density of the active nucleation sites, and the improved boiling surface wettability. Also, the effects of adding a polymer into the working fluid on the nucleate boiling heat-transfer capability depend on the polymer used. The polymers may improve the boiling HTC below the optimum concentration or degrade the heat-transfer behavior, depending on changes in the viscosity of the fluid. While the enhancement effect is due to the reduced bubble coalescence and enhanced nucleation, the degradation can be attributed to the decrease in vapor bubble growth rate, the contact angle decrease, or the suppression of microconvection. The main concerns associated with the addition of surfactants and polymers are degradation over time, strong incipience excursion, and environmental impact.

## 4. Self-Rewetting Fluids

Many authors have focused their research on enhancing the nucleate pool boiling heat-transfer capability by altering the transport characteristics of the employed working fluids. Despite the fact that the surface tension of most liquids presents a decreasing trend with a rising operating temperature, exceptions can be observed, with surface tension enhancing with rising temperature. Such surface tension tendency facilitates the thermocapillary flow from the cold to the hot regions of the liquid–solid interface.

The self-rewetting fluids were introduced by Abe [99], who examined the heat-transfer ability of dilute aqueous solutions of high-carbon alcohols. The alcohol-rich components of such solutions evaporate during the fluid-to-vapor phase change, conducting to the presence of a concentration difference in the fluid–vapor interface, which in turn conducts to a surface tension difference. Also, the temperature gradient that is created in the fluid–vapor interface also contributes to the surface tension difference. In addition, the Marangoni flow caused by the concentration difference along with the thermocapillary flow induces a vigorous liquid inflow at the three-phase contact line of the bubble and heating surface. It is known that the fluid flow naturally to higher temperature regions and, hence, the concept of self-rewetting fluids originates from that spontaneous liquid supply to the dry or hot patches; this effect, coupled with the Marangoni flow, will cause a CHF increase. The alcohol aqueous solutions, presenting a chain length longer than four carbon atoms, provide extra pool boiling heat-transfer enhancement, as derived from their surface tension.

**Figure 20:**
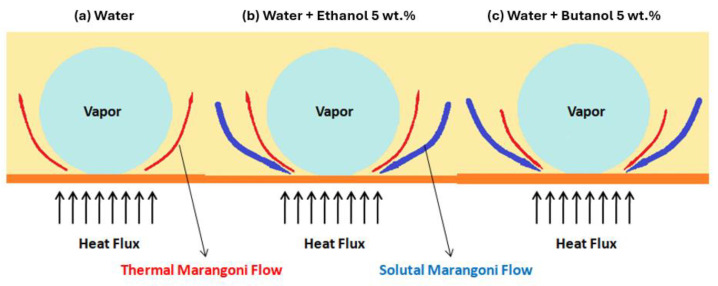
Schematic illustration of the nucleate pool boiling mechanisms for (**a**) water, (**b**) water and ethanol 5 wt. %, and (**c**) water and butanol 5 wt. % Adapted from [88].

Furthermore Zhou et al. [100] investigated the deionized water nucleate boiling and the nucleate boiling of the self-rewetting fluids n-butanol, n-pentanol, and n-hexanol aqueous solutions in spherical glass beads packed-bed porous structures. The results indicated that the HTC decreased with increasing heat flux at low-heat fluxes until it reached a value that only suffered a minor change, and the HTC increased with an increasing heat flux of up to 35 kW/m^2^. It was also shown that the HTC increased with increasing amounts of carbon in the self-rewetting solutions. Figure 20 schematically illustrates the mechanisms involved in the nucleate boiling process using self-rewetting fluids.

Zupancic et al. [101] carried out the pool boiling of n-butanol and n-butanol and water mixtures on thin titanium foil and employed synchronous high-speed infra-red and video cameras to observe the transient fields of temperature and the vapor bubbles’ dynamics. The self-rewetting fluids at the onset of nucleate boiling provided an increased density of active nucleation sites and smaller bubble diameters in comparison to those obtained with only water. Nevertheless, with growing heat flux, a strong depletion of n-butanol was generated in the vicinities of the active nucleation sites, causing periodic fluctuations in the temperature of nucleation and vapor bubble evaporation energy. When using the self-rewetting fluids, the mean temperature of the boiling surface and the vapor bubble diameter at the detachment form the surface stage were superior to those achieved with water alone. Such facts indicated that the Marangoni effect cannot overcome the limited mass diffusion of more volatile components toward the active nucleation sites in the flat horizontal heating surfaces’ pool boiling processes.

Based on the authors observations, a self-rewetting working fluid will not attain a boiling HTC superior to that of the water, which is also known for conventional mixtures with negative temperature-dependent surface tension. The pool boiling tests were elaborated on a horizontally oriented, Joule-heated titanium foil under terrestrial gravity using water and two concentrations of self-rewetting n-butanol-water mixtures. High-speed video and IR cameras were used to visualize the bubble’s life cycle and the transient temperature fields of the boiling surface at two different heat fluxes, i.e., 40 kW/m^2^ and 80 kW/m^2^. On average, the self-rewetting fluids did not significantly enhance the nucleation frequency or the replenishment of active nucleation sites, or decrease the bubble’s departure diameter to obtain smaller thermal variations on the boiling surface.

When dealing with only a single isolated nucleation site, the 6% n-butanol solution showed periodic fluctuations of the departure diameter of the vapor bubbles, temperature of nucleation, and energy removed by a single bubble due to microlayer evaporation. The results indicated that the localized concentration variations dictated the bubble dynamics, even during the early stage of the nucleate boiling process. Despite the favorable Marangoni flows provided by the self-rewetting fluids (benefiting the heat-transfer behavior in heat pipes, flow boiling, and pool boiling with one-dimensional heaters such as wires), the results on a flat heater did not show the same. The solutal and thermal Marangoni flows cannot overcome the limitations of mass diffusion in the vicinities of the active nucleation sites. Because of this, the HTC at low-heat fluxes did not increase by employing the self-rewetting fluids when compared to the one obtained with water. Nonetheless, the boiling tests using thin surfaces and synchronous high-speed thermography and video camera offer the opportunity to investigate the bubble dynamics and localized heat transfer mechanisms together with the localized concentration variations in the vicinities of a single nucleation site through the measured nucleation temperature and bubble diameter.

Moze et al. [102] evaluated the pool boiling heat-transfer performance using water and self-rewetting mixtures of water and 1-butanol on hybrid functionalized aluminum surfaces. The microstructured surfaces were produced via chemical etching in hydrochloric acid and the cavity mouth diameters, ranging from 3.6 μm to 32 μm, led to an optimized effective bubble nucleation and provided a superior boiling performance. Extended etching periods of 10 and 15 min led to uniform corrosion; additionally, the prepared super-hydrophilic surfaces had a micropeak structure that lacked surface microcavities for more effective nucleation. In the second stage, the surfaces combining lower surface energy and a modified surface microstructure were produced via the hydrophobization of etched aluminum surfaces, employing a silane agent.

The authors experimentally verified that the hydrophobized surfaces improved the boiling heat-transfer performance by having boiling curves with a considerably lower superheat. Significant heat-transfer enhancement was observed for hybrid microcavity surfaces with a low surface energy. The synergistic effect of the enhanced boiling surfaces and a self-rewetting fluid was not observed. An improved CHF was only obtained for the untreated surface, while a lower CHF and lower HTCs were measured on functionalized surfaces, whose properties were already tailored to promote nucleate boiling. A stepwise approach to surface modification was successfully demonstrated through the development of hybrid functionalized surfaces. A short period of chemical etching treatment induced pitting corrosion, creating surface microcavities with optimal diameters for nucleation under pool boiling conditions.

The evaluation of the boiling performance of the etched surfaces using pure water confirmed that the surface functionalization using short etching times, producing a microcavity surface morphology, was preferable to the prolonged etching periods that formed a micropeak surface morphology, lacking the nucleation-promoting surface features such as the vapor trapping surface cavities. The hydrophobization of the microcavity and micropeak heat transfer surfaces demonstrated that an extra amelioration of the heat-transfer behavior could be attained by decreasing the surface energy. The best boiling performance was achieved with a hydrophobized microcavity surface in which the HTC reached 305 kW/(m^−2^·K^−1^), representing an enhancement of 488% in comparison to an untreated surface, whereas the CHF remained close to that of an untreated heating surface. Furthermore, complete transition into nucleate boiling is achieved at a very low superheat of around 1.1 K, allowing for efficient heat dissipation, even at low-heat fluxes.

The results on hydrophobized etched surfaces demonstrated that tailoring the microstructure of the surface to promote the bubble nucleation and the surface energy to reduce the energy barrier for the onset of nucleate boiling had a concomitant benefit for the boiling thermal performance. The boiling of self-rewetting fluids on the untreated reference surface provided an increased CHF, decreased bubble diameters, increased nucleation frequencies, and limited bubble coalescence compared to what happened in the water pool boiling. However, for both tested functionalized surfaces, the HTC deteriorated, and the CHF decreased relative to the boiling performance obtained with pure water. The boiling of self-rewetting fluids, therefore, did not enhance the heat-transfer capability on the surfaces, which were tailored to promote nucleate boiling, as bubble diameters and coalescence were already reduced through surface functionalization. Therefore, an additional reduction in bubble diameter induced by the self-rewetting fluid resulted in a deteriorated heat-transfer performance for both the microcavity surfaces.

In sum, the origin of the distinct heat-transfer behaviors between the self-rewetting fluids and other heat-transfer fluids, like water and its mixtures and mixtures of fluids, derives from the capillarity effect, the Marangoni effect, and the specific wettability inherent to the contact angle and surface tension. Generally, the self-rewetting fluids exhibit better heat-transfer characteristics than their water base fluid. Taking the example of the n-butanol, the increase in the n-butanol concentration will enhance the thermal performance of the fluid in comparison to the one attained with the water alone. Nonetheless, the optimum concentration value is not evident, given that the growing n-butanol concentration improves the wettability but deteriorates the surface tension and thermophysical characteristics of the heat-transfer fluid. To clarify this issue, further experiments should be undertaken testing different concentrations of n-butanol aqueous solutions to identify the optimal concentration that may achieve the best nucleate pool boiling heat-transfer performance. The same applies to other self-rewetting fluids.

## 5. Nanofluids

Nanofluids have attracted great interest from the research community; these are heat-transfer fluids containing nanoparticles dispersed in a base fluid. The nanoparticle materials include, among others, metals, metal oxides, and different forms of carbon. Two of the most relevant factors for the synthesis of nanofluids are their stability and uniform dispersion, which can be attained through different methods, including adjusting the pH of the suspensions to keep the nanoparticles far from their isoelectric point, the application of ultrasonic vibration, and incorporating surfactants and dispersants.

**Figure 21:**
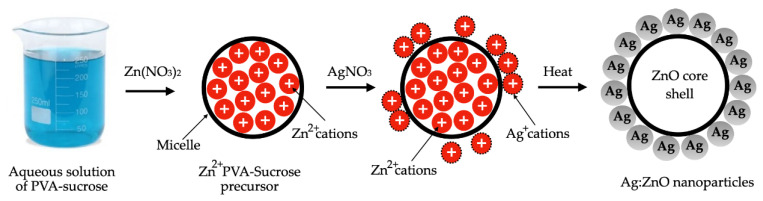
Schematic diagram representing the synthesis of the hybrid silver–zinc oxide nanoparticles. Adapted from [103].

Sharma et al. [103] examined the pool boiling heat-transfer enhancement of a silver–zinc oxide nanofluid with concentration values ranging between 0.02% and 0.1% vol. A cylindrical copper material measuring 20 mm in diameter was employed as the boiling surface. The thermal conductivity of the hybrid nanofluids was reported to be 32% higher at 45 °C in comparison to that obtained with deionized water. During the pool boiling experiments, CHF and HTC peak values of 65.5% and 179.9%, respectively, were reached at 0.1% vol., in comparison to those achieved with deionized water only. The authors also confirmed that any volumetric concentration of the nanofluids beyond 0.1% vol. decreased the boiling heat-transfer ability of the system. Figure 21 presents the schematic diagram of the preparation method of the hybrid silver–zinc oxide nanoparticles.

As the main conclusions, it can be stated that the developed hybrid nanofluids revealed high CHF values in the pool boiling process in comparison to their traditional counterparts, fundamentally comprising nanofluids with individual nanoparticles; hence, the exploration of their use in high-heat flux applications is enabled. These enhancements derived essentially from enhancement in the available heat transfer surface area and the density of active vapor nucleation sites. It should also be highlighted that the boiling curves of the hybrid nanofluids shifted to the left in reference to that obtained with deionized water. This is evident for all the tested nanofluid concentrations. It is also noteworthy that the horizontal gap between the adjacent boiling curves decreases with rising nanofluid concentration, indicating the existence of an upper limit for the concentration of nanofluids.

Liu et al. [104] studied the nucleate pool boiling performance of aqueous nanofluids containing multi-walled carbon nanotubes measuring 15 nm in diameter and 5 µm–15 µm in length, with weight fractions ranging between 0.5% wt. and 4% wt., and under pressure values ranging between 7.4 kPa and 103 kPa; here, the carbon nanotubes were treated with nitric acid to promote their uniform dispersion in the base fluid. The carbon nanotube nanofluids enhanced the HTC and CHF considerably compared to the water itself, and the enhancement improved significantly with decreasing pressure. For all pressures tested, the maximum nucleate boiling heat-transfer enhancement was achieved with 2 wt. % of carbon nanotubes. The researchers also performed pool boiling experiments with pure water on a carbon-nanotube-coated heating surface for comparison; they found that carbon nanotube nanofluids provide better enhancements of CHF and HTC.

Kamel and Lezovits [105] experimentally determined the pool boiling behavior of a copper heating tube using aqueous cerium oxide nanofluids. The boiling curves and pool boiling HTCs for deionized water and nanofluids at atmospheric conditions were determined. The nanoparticles were diluted into deionized water according to volumetric concentration values between 0.001 vol.% and 0.04 vol.% The obtained results confirmed that the HTC for the water and nanofluids were augmented by the growing imposed heat flux; this fact was clear from the generation of vapor bubbles during the nucleate boiling regime. Additionally, the boiling HTC of the synthesized nanofluids increased for all the experimental concentrations; a more pronounced increase was found for a nanofluid concentration of 0.007 vol.%, which was about 1.7 using the HTC enhancement ratio (HTC of nanofluid/HTC of water) in comparison to that of the deionized water at low-heat fluxes.

The HTC and pool boiling curve using deionized water and the horizontal tube made of copper with a moderate roughness were both validated against correlations in the literature and showed good agreement. It was verified that, with the use of aqueous cerium oxide nanofluids at 0.007 vol.%, the superheats at low/moderate/high-heat fluxes could be reduced, promoting the shifting of the boiling curves to the left and enhanced the boiling HTC. The higher HTC ratios for cerium-oxide-based water nanofluids were about 1.7 and 1.6 for volume concentrations of 0.007% and 0.004% at low-heat flux, respectively. This result can be used to argue that this enhancement could be associated with the bulk and surface modification when using these nanofluids and boiling surface geometry.

The alteration of the surface properties, including capillary wicking, wettability, and roughness, had a profound impact on the pool boiling thermal performance. The enhancements in the surface roughness and thermal conductivity of nanofluids improved the pool boiling thermal performance for diluted concentrations, specifically at low- and moderate-heat flux values. The adherence of the nanolayer on the surface because of the deposition of the nanoparticles was found to be poor at high-heat fluxes with this surface morphology; this could be attributed to the mechanism of the generation of the vapor bubbles around the horizontal tube (sliding the bubbles from the tube sides). This could be the fundamental reason behind the enhancement at low and moderate heat fluxes with low-concentration nanofluids.

**Figure 22:**
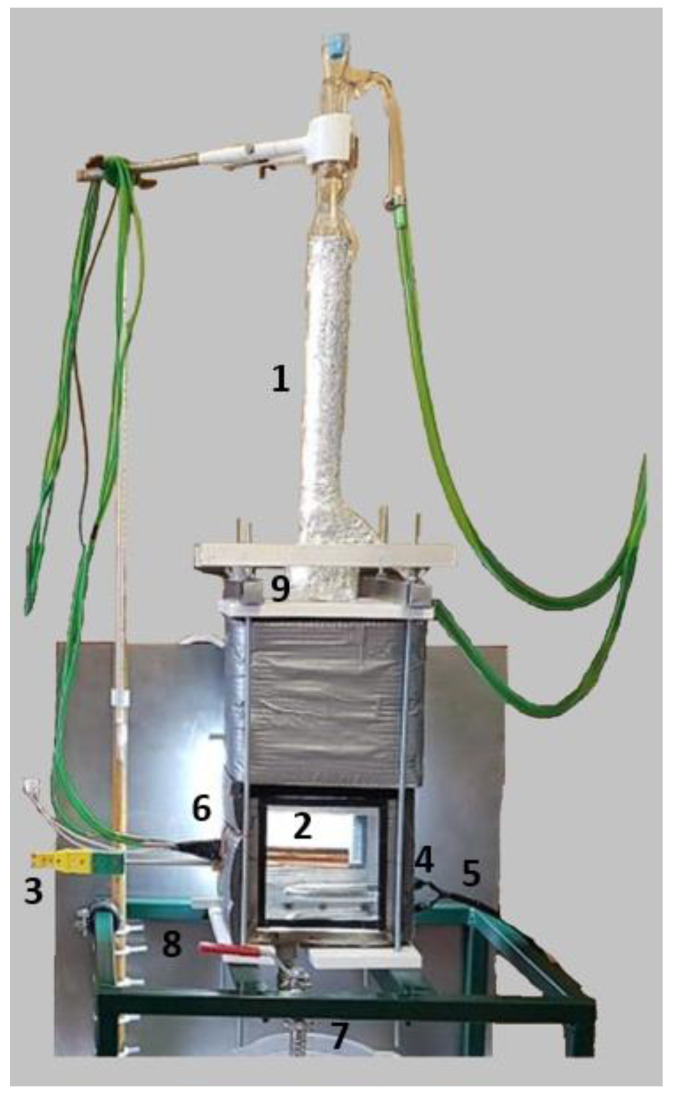
Pool boiling setup: 1—cooling condenser; 2—copper heating tube; 3—K-type thermocouples; 4—auxiliary heater; 5—autotransformer; 6—autotransformer; 7—drain valve; 8—temperature logger; 9—injection valve. Adapted from [105].

**Figure 23:**
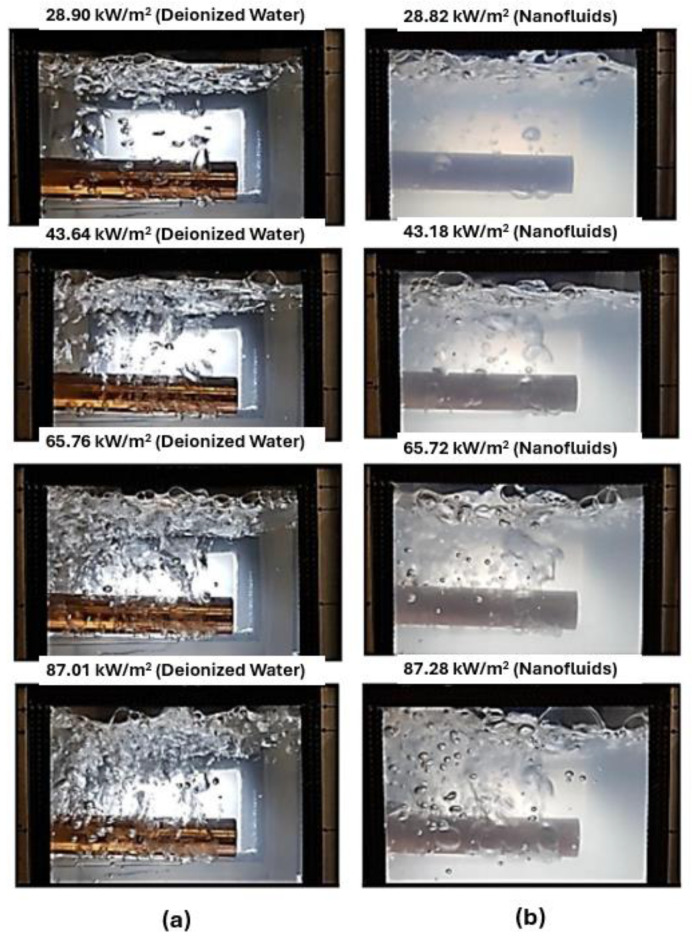
Images of the bubble dynamics during the pool boiling of (**a**) deionized water and (**b**) cerium oxide nanofluids at 0.001 vol.% and diverse heat fluxes [105] (unrestricted use: publication license from the authors is not required).

Also, the authors observed that the size of the bubbles increased with growing imposed heat flux using water and nanofluids, but the growth was faster in water in comparison to the nanofluid at larger heat fluxes, leading to more towering and uneven columns of vapor bubbles. Figure 22 shows the pool boiling setup that was used. Figure 23 shows images of the bubble dynamics during the pool boiling process.

Furthermore, Park et al. [106] argued that the HTC for aqueous nanofluids with carbon nanotubes measuring between 10 nm and 20 nm in diameter and between 10 and 50 µm in length, at volumetric fractions from 0.0001 vol.% to 0.05 vol.%, was inferior to that for water. The deterioration in the HTC was attributed by the researchers to the heating surface deposition layer that acted as thermal resistance and decreased the population of active nucleation sites. Nonetheless, the CHF for the nanofluids with carbon nanotubes increased with the growing concentration of nanotubes due to the improved wettability of the surface, achieving a peak enhancement of 200% at 0.001% vol.; beyond this, the CHF enhancements were reduced due to the considerable conglomeration of the carbon nanotubes.

In accordance with this, White et al. [107] attempted to isolate the effects of the deposition layer from those of the suspended nanoparticles. This was accomplished by conducting pool boiling experiments on a surface, alternating between water and a water-based 2.3 vol.% zinc oxide nanofluid. The authors found that the HTC for water was augmented by the growing number of experiments, which was attributed to the gradual increase in the surface roughness of the deposition layer. The results for the nanofluid were less monotonic, as the HTC increased at first as compared to water alone, before the deposited layer completely formed; this enhancement was attributed to the thermophysical properties of the nanofluid. However, once the layer fully covered the surface, the HTC steadily diminished due to the bubble nucleation suppression and bubble motion through the suspended nanoparticles.

Overall, the following considerations on the nucleate pool boiling of the nanofluids should be highlighted: (i) Most of the published studies provide evidence of nanoparticle deposition onto the boiling surface. In fact, most of the experiments were performed with stable nanofluids over only a short period, and the long-term behavior was usually excluded from the experimental works.

Concerning this, Lee et al. [108] reported long-term variations in the pool boiling performance by investigating the CHF enhancement and the long-term stability of water-based magnetite nanofluids. Hence, the long-term stability of the nanofluids is a relevant concern, particularly for applications demanding unaltered long-term thermal management performance. (ii) One of the problems concerning the use of nanofluids is the potential clogging of flow features and passages of the heat transfer devices and systems, given that the nanoparticles can agglomerate into larger clusters that may cause operation failures. (iii) The nanoparticle deposition of nanoparticles onto the boiling surface during the nucleate pool boiling evolutes in time, which is put together with the decrease in the concentration of the nanoparticles in the bulk liquid. Both phenomena may have an impact on the nucleate boiling behavior. Additionally, the temporal performance variations over extended periods might compromise the thermal management reliability in most applications. (iv) Certain large-scale applications may entail the use of considerable quantities of nanofluid, which would appreciably increase the thermal management cost. In addition, there is the extra cost of the preparation methods of the nanofluids in the pursuit of fine, uniform dispersion and the improved stability of the nanoparticles in the base fluid. (v) Most researchers acquire nanoparticles from different vendors and batches, which can provoke variations in the characteristics of the nanoparticles, including size and size distribution, thermophysical properties, and purity; such variations may affect the consistency of the available nucleate pool boiling data for the same type and concentration of the nanofluids. (vi) There is evidence in the literature to suggest that the surface treatments based on the deposition of nanoparticles through nanofluid boiling entails the risk of particle detachment, which raises concerns surrounding the long-term sustainability of the heat-transfer performance.

**Figure 24:**
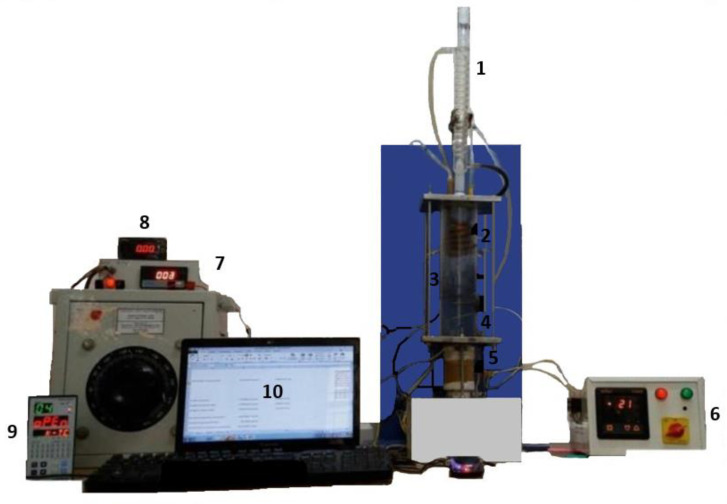
Pool boiling setup: 1—reflux condenser; 2—copper condenser; 3—borosilicate glass; 4—auxiliary heater; 5—copper heater; 6—thermostat; 7—autotransformer; 8—ammeter; 9—data logger; 10—data storage PC. Adapted from [109].

In addition, Reddy and Venkatachalapathy [109] focused on the amelioration of the pool boiling thermal performance and CHF by mixing nanoparticles of alumina and copper oxide at different concentrations in deionized water at concentrations ranging between 0.01% vol. and 0.1% vol. The thermal conductivity of the hybrid nanofluids was increased nearly up to the maximum of 15.7% at 0.1% vol. and at room temperature when compared with that of the deionized water alone. The CHF of hybrid nanofluids was larger than that of the single-type nanofluid in the pool boiling experiments. The boiling curves of hybrid nanofluids shifted towards the left in comparison with those of the deionized water. With increasing concentrations, the curves shifted towards the right due to particle deposition, leading to enhanced capillary action. A peak enhancement of around 49.8% in the CHF was obtained at 0.1% vol. volumetric concentration. The boiling HTC was increased by 7.1% and 6.7% at 0.01% vol. and 0.03% vol., respectively, in comparison to those obtained using only deionized water; they started to deteriorate with increasing volumetric concentrations due to the rising deposition of nanoparticles onto the heating surface. Figure 24 illustrates the developed pool boiling setup.

Overall, the nanofluids offer enhancing trends on the CHF, which originated in the ameliorated surface wettability through the nanoparticle deposition during boiling. Nonetheless, there are still contradictory facts related with the impact of nanofluids on the HTC derived from parameters like the fluid type, applied heat flux, the roughness of the boiling surface, type, dimensions, and concentration of the incorporated nanoparticles, and the preparation and functionalization methods. Also, there are still many concerns inherent to the exploration of nanofluids in boiling conditions and in thermal management equipment like clustering, sedimentation, and precipitation of nanoparticles, clogging of flow passages, erosion to boiling surfaces, nanoparticle deposition during the boiling process, and transient heat-transfer behavior. Apart from these, there are the general problems associated with the preparation of the nanofluids, such as their elevated overall cost, difficulties in their production, and the lack of quality assurance in their scalability. Despite all difficulties, nanofluids are very promising for use in CHF enhancement.

## 6. Influence of the Pressure of the System

Regardless of the used pool boiling enhancement technique, the pressure of the system should always be taken as a game-changing factor in pool boiling conditions. First of all, the pressure affects the thermophysical properties of the heat-transfer fluids; this is because, for example, the increasing pressure reduces the latent heat of vaporization and the specific volume of water. As the property of the liquid changes with the increasing pressure, the size of the departing bubbles changes. The results suggested that a change in the specific volume of the vapor may cause the change in the bubble departure size. A direct correlation was also found for the nucleation site densities with pressure. A significant enhancement in the performance of low-pressure pool boiling has also been reported [110]. There is not enough data to explain the complexity involved in the high-pressure pool boiling process. Pressure activates the nucleation sites at micron size of cavities. For example, at the same wall of superheat at 10 K, the size range of active nucleation sites is much larger than the atmospheric pressure. Henceforth, we obtain more nucleation points on the heating surface.

Sakashita et al. [111] studied the boiling behavior on a horizontal plate in a saturated pool of boiling water at pressures up to 701 kPa. The researchers found that the pressure of the system influenced the size of the nucleating vapor bubbles, but the detachment frequency of the coalesced bubbles was unaffected. They also observed that the diameter of coalesced bubble increased with the heat flux and pressure. The research team also found that the size of the departing bubble was inversely proportional to the pressure, leading to coalescence at very-low-heat flux regions. Labuntsov et al. [112] investigated the similar nature in the growth rates of departing bubbles at high pressures. They analyzed their studies based on the evaporation rate of liquid close to the bubble base. Indeed, at high pressures, the excess rate of change of the enthalpy of the bulk liquid surrounding the bubbles impacts the bubble size. They performed many experiments at high pressures on horizontal and vertical surfaces, and found that the pressure slows down the bubble growth rate. It was also demonstrated that the bubble departure frequency increases with increasing pressure. With increasing pressure, the surface tension of the liquid decreases, which may affect the reaction forces on the departing bubbles. At high pressure, the detachment frequency increases.

Sakashita [113] observed the boiling behaviors on a horizontal, upward-facing plate with atmospheric pressures up to 7 MPa. The authors observed only a negligible effect in the detachment frequency at high pressures and heat fluxes. The pressure effect can be observed on the nucleation site densities. A different nucleation behavior was noticed at high pressure. Sakashita et al. [111] made a similar observation on the number of active nucleation sites. They carried out a systematic study for the nucleation site densities on the pressure under one heat flux condition and reported the nucleation density increases in proportion of 1.5th of the pressure. The influence of the pressure can be seen on the degree of superheats. The degree of superheat decreases with the growing pressure. This could be due to a change in the hydrodynamic behavior of the departing vapor bubbles. Before nucleate boiling, most of the heat transferred to the liquid is due to natural convection. The formation and coalescence of the vapor bubbles may impact the thermal boundary layer around the contact line of the bubbles, which would impact the boiling behavior. A majority of the published results show that the pressure improves considerably with heat-transfer performance.

Guan et al. [114] carried out pool boiling experiments at pressures from 150 kPa to 450 kPa on a brass boiling surface using different heat-transfer fluids. In the natural convection regime, the authors noticed that there was no considerable impact of the pressure on the HTC. Nonetheless, the researchers noticed a remarkable effect of pressure in the HTC at high-heat fluxes. Moreover, Li and Betz [115] reported a 126.8% enhancement in the HTC at atmospheric pressure and 51.5% at 308 kPa.

Daharya and Betz [116] conducted an experimental work to evaluate the effect of pressure on the heat-transfer enhancement during the pool boiling of a plain copper surface. The experiments were conducted at different pressures from 0 kPa to 413.7 kPa. The onset of nucleation occurs at different superheats for different pressures. At heat fluxes below 250 kW/m^2^, the boiling performance is not significantly affected as compared to the high-heat flux. The variation in the degree of superheat is larger at low-heat flux as compared to the high-heat flux. At high-heat fluxes, a considerable difference in the wall superheat degree was verified. In the nucleate boiling region, a change in the bubble dynamics strongly affects the slope of the boiling curve in becoming steeper with increasing pressure. At high pressure, a transition in the boiling curve is noticed after 250 kW/m^2^. It could be the reason a significant change is noticed in the HTC at high-heat flux compared to the low-heat flux. At 1000 kW/m^2^, 50%, 75%, 125%, and 175% enhancements in the HTC were observed in comparison to those of the 0 kPa for 103.4 kPa, 206.6 kPa, 310.2 kPa, and 413.7 kPa, respectively. At an imposed heat flux of 250 kW/m^2^, the HTC changed from 20 to 40 kW/m^2^.K, with the pressure varying from 0 kPa to 413.7 kPa. The obtained results suggested that pressure has a strong impact on the heat-transfer performance at high-heat flux. Also, it was found that the CHF increased with increasing operating pressure. CHF values of 1100 kW/m^2^, 1200 kW/m^2^, 1220 kW/m^2^, 1300 kW/m^2^, and 1420 kW/m^2^ were reported at 0 kPa, 103.4 kPa, 206.6 kPa, 310.2 kPa, and 413.7 kPa, respectively.

A similar trend was found by Li and Betz [103]. At 0 kPa, the bubble starts nucleating at 18.570 kW/m^2^. The departing bubble diameters were measured as approximately 4.58 mm, 2.42 mm, and 2.37 mm at 0 kPa, 413.7 kPa, and 620.5 kPa, respectively. The pressure reduced significantly the size of the nucleating bubbles. Around 46% of reduction was observed in the bubble size from the atmospheric condition to the maximum 620.5 kPa. The main findings were that the pressure had an impact on the bubble departure size, the detachment frequencies, and the nucleation site densities. The maximum heat-transfer enhancement was found to be about 175% at 413.7 kPa. The experimental results suggested that pressure enhances the heat-transfer enhancement.

## 7. Limitations, Challenges, and Recommendations for Future Research

Some recommendations and guidelines for further research topics on the challenging pool boiling enhancement area of research are summarized as follows:

(i) Despite the improvements in the nucleate pool boiling heat-transfer performance through innovative coating materials and techniques, large-scale applications of these concepts remain hindered or even unpractical. To resolve this limitation, better investigations of the cost-effectiveness and longevity of the coatings should be given priority; these are the fundamental parameters that still limit industrial implementations in applications like self-cleaning, aerospace water harvesting, and thermal management, based on the use of heat exchangers, thermosyphons, and other devices. In this sense, novel fabrication methodologies should be implemented to ensure the reliability, cost-effectiveness, and longevity of the enhanced surfaces. Although effective methodologies have been reported, there remains a lack of comprehensive techno-economic analysis for the enhanced heating surfaces to be applied in practical situations.
(ii) A ranking criterion for the structured surface enhancements in the CHF and HTC under pool boiling scenarios should be defined. A minimum percentage in comparison to a plain heating surface in which the enhancement qualifies the surface should be defined as possessing superior HTC and CHF. It would be also useful to classify the techniques for heat-transfer enhancement according to the findings from standardized methods under the same conditions.
(iii) The published results often demonstrate the benefits of biphilic surfaces over the conventional hydrophilic and hydrophobic surfaces. However, there are still many unknown aspects related to this technology that should be further clarified. For instance, the durability of the hydrophobic regions should be further investigated to assess whether they can withstand long-term continuous droplet formation and departure. Also, the shape, size, and pitch of the wettability patterns need to be optimized for heat-transfer enhancement purposes.
(iv) The inconsistent published findings and results related to the pool boiling HTC using nanofluids by means of improvement or deterioration can be mainly attributed to the surface modification effect. When the surface interaction parameter, defined by the ratio between the size of the nanoparticles and the surface roughness, is greater than one, the likelihood of great new cavities is higher. Therefore, new sites for nucleation and growth of the vapor bubbles become more elevated, especially with low concentrations.
(v) It is recommended that, when exploring the nanofluids pool boiling, efforts should be made to select the most suitable size of the nanoparticles according to the original roughness of the heat transfer surface, particularly when dealing with tubes and flat-plate heating surfaces.
(vi) More studies on multiscale enhancement techniques should be conducted to facilitate the liquid supply and improve the bubble dynamics to further ameliorate the pool boiling thermal performance. Examples of this are the super-biphilic boiling surfaces with enhanced pool boiling performance; this should be used to effectively dissipate energy under the form of heat and avoid thermal failure in high-power devices and systems.
(vii) More experimental and numerical works on the suitability for pool boiling enhancement of bi-conductive heat transfer surfaces are most welcome. When using this methodology, the thermal performance improvement derives from the bulk thermal conductivity instead of the surface structures and properties. In contrast to surface coatings, this boiling enhancement technique is not susceptible to material degradation and long-term reliability concerns. Nor is it reliant on specific fluids or fluid properties to promote boiling, as it is needed for low-surface-energy material surfaces.
(viii) The theoretical analysis of the enhancement of pool boiling heat transfer, especially for advanced empirical correlations, should be further improved. It is necessary that we implement formulas for specific models to estimate the results in a much more accurate manner. Also, the existing numerical simulations on the subject are rather scarce and sometimes inconclusive. Hence, further conclusive numerical studies are highly recommended.
(ix) The boiling surface coating with nanoparticles and the use of nanofluids (nanoparticle deposition) is one of the most-employed modes to modify the heat transfer surface. The nanostructured layer surfaces are capable of effectively enhancing the pool boiling CHF and HTC because of the enhanced roughness, the density of active nucleation sites, and the capillarity effect. Nonetheless, the main related concern is that the service lifespan of those surfaces is very limited since, for instance, the nanoporous surfaces might degrade or even peel off after many boiling cycles. Hence, it is strongly suggested that we attempt to extend the pool boiling lifespan of such heating surfaces. Also, for nanowire-, nanofiber- and nanotube-coated surfaces, the published works are comparatively rare and further research studies on the matter are recommended.

## 8. Conclusions

This work provides a comprehensive survey of the published articles addressing surface modification and the use of innovative fluids for the enhancement of nucleate pool boiling heat transfer. The current work also addressed the fundamental limitations and challenges that are closely related with the implementation of the heat-transfer enhancement methodologies in thermal management systems and equipment. The main findings of the current review are summarized as follows:

(i) The implementation of structured enhanced surfaces for pool boiling situations involves concerns that are intertwined from several points of view. On the one hand, the structures entail surface irregularities and enhance the number of active bubble nucleation sites, which can improve the heat-transfer ability. On the other hand, and considering the Cassie–Baxter theory, the structures alter the wettability and contact angle which regulate the interfacial hydrodynamic behavior of bubbles, promoting the initiation of the CHF. Hence, the role of the surface structures and surface wettability in altering the vapor bubble generation and rate of heat transfer needs further in-depth investigation.
(ii) Experimental CHFs are usually far less than the theoretical upper limit by 2–3 orders of magnitude, suggesting that the technique of boiling heat transfer possesses large space for improvement. The boiling surface modification is an effective and commonly employed route to increase the nucleate boiling heat-transfer capability. Extending the effective heat transfer surface area, tuning the surface wettability, increasing the number of active nucleation sites, and activating the capillary wicking effect, among others, are normally considered for pool boiling performance enhancements.
(iii) The enhanced structured boiling surfaces comprise nanostructured and microstructured surfaces. Many techniques such as numerical control machining, wire electric discharge machining, laser ablation, lithography, and plasma, among others, are commonly chosen to synthesize microscale surface structures, including cavities, grooves, fins, channels, and pillar arrays. These structured boiling surfaces will enhance the surface roughness, the density of active nucleation sites, the effective heat transfer surface area, and the surface wicking ability, and will also separate the liquid and vapor pathways to aid the growth of the vapor bubbles and their departure from the surface. All these factors will make the nucleate pool boiling behavior easier and considerably improved. Nonetheless, the elevated fabrication costs and complex manufacturing processes limit the promotion and application of such surfaces. In addition, as analyzed above, the constant properties indicate they usually perform well at specific boiling conditions.
(iv) The surface roughening (Section 2.7) and the use of fins, microchannels, tunnels, reentrant cavities, and microporous structures, among others, have their main beneficial features in the increased density of active nucleation sites that increment the nucleation boiling HTC. However, the effect of these techniques on the CHF varies widely. Also, the use of microporous structures in the boiling surface can be particularly noticeable given their capability to enhance the CHF through the vapor–fluid pathways separation.
(v) The coating of the heating surface with nanoparticles, nanofibers, nanowires, nanotubes, and nanoporous and nanofilm layers, among others, possess the benefit of a nucleate boiling heat-transfer enhancement by means of capillary wicking within the nanostructures. The taller nanotubes often give an increased CHF and HTC. The underlying mechanism for such finding is not yet completely understood, but the presence of vapor embryos at low-heat fluxes and pathways for fluid flow appear to be accountable. However, the topography of these nanostructures may provoke blockage, causing enhanced decay over time.
(vi) The nanoscale surface enhancements based on the use of nanotubes, nanowires, nanofibers, and nanoparticles (Section 2.1) are carried out through chemical synthesis or self-assembly approaches, being scalable, simple, and cost-effective, and they can be applied with diverse metals, metal oxides, and other materials. Nonetheless, the wide distribution of feasible sizes, shapes, and spacings in the nanoparticles and nanotubes remains a demanding challenge. In addition, the strength of the interface between the nanoparticles and the boiling surface can be not enough to maintain the harsh environment provoked by the working fluid or vapor bubbles at the high temperatures inherent to the pool boiling process; this leads to the eventual delamination of the films and nanoparticle deposition layer damage or detachment.
(vii) The porous structures in the pool boiling surfaces (Section 2.2) increase the spreading of the fluid under the vapor conglomerates, conducting the rewetting of the dry regions in the pre-crisis mode and CHF enhancement during the pool boiling process. Also, the nanoporous structures offer many challenges for their use in pool boiling like, for instance, their preservation in time and consequent loss of heat-transfer performance after prolonged pool boiling experiments.
(viii) The pool boiling surfaces with reentrant cavities and structures (Section 2.4) can strongly contribute to the enhancement of pool boiling heat transfer. The reentrant cavities are commonly recognized to act as vapor traps during the nucleate pool boiling process. Such vapor traps considerably facilitate the bubble nucleation, enhance the heat-transfer ability between the operating fluid and the heating surface, and delay the occurrence of CHF.
(ix) The grooved pool boiling surface structures (Section 2.5) enhance the surface roughness, increase the effective heat transfer surface area, increase the density of the bubble nucleation sites, and enhance the wicking effect, resulting in considerably improved boiling performances. They also separate the liquid and vapor pathways to facilitate the generation of vapor bubbles and their departures from the surface. The grooved surfaces are effective in enhancing the pool boiling heat transfer, given that the grooves provide nucleation sites and act as conduits for liquid flooding caused by bubble lifting and/or capillary wicking, improving both phase-change heat transfer and convection heat transfer. The size of the grooves impacts the nucleation of bubbles, liquid and vapor separation, and liquid entrance length.
(x) The biphilic wettability boiling surfaces (Section 2.6) combine the beneficial features of the homogeneous hydrophilic and hydrophobic surfaces to further improve the pool boiling heat-transfer capability. This type of heating surfaces exhibits developed heat-transfer enhancement tendencies and the performance of the pool boiling process is usually ameliorated at low-high wall superheats.
(xi) The pool boiling process exploring metal foams (Section 2.9) usually presents a considerable heat-transfer enhancement in comparison to that attained with the plain boiling surfaces. The foams promoted a higher CHF and HTC and a decreased wall superheat at the onset of the nucleate boiling. The main advantages of the foams are the increased heat transfer area, fluid disturbing, and the wetted area that facilitates the generation of vapor bubbles at low-heat fluxes together with an increased density of active nucleation sites at high-heat fluxes. The main parameters of the foams are their density of the pores and thickness. The wet area augments with increasing density of pores. An increased pore density will make the pore diameter decrease. The small pores augment the capillary-driven liquid flow towards the foam. Nonetheless, the small pores prevent the bubble departure at high-heat fluxes. Also, thicker foams exhibit enhanced wetted area and increase the HTC at low-heat fluxes, whereas the same thicker foams degrade the nucleate boiling heat-transfer performance at high-heat fluxes because of the thermal resistance produced by the vapor trapped in the porous structure.
(xii) The conductive boiling surfaces (Section 2.10) are composed of a high-temperature region presenting active nucleation sites for vapor bubble generation and a constantly wet region, promoting the supply of the heat-transfer fluid to the boiling surface. The bi-conductive heating surfaces are a very suitable way for pool boiling heat-transfer enhancement, particularly at high-heat fluxes. Also, the bi-conductive surfaces involve no susceptible coatings or structures and usually have the beneficial features of an enhanced longevity, structural strength, and reduced heat-transfer behavior degradation in time.
(xiii) The addition of surfactants (Section 3) into the boiling heat-transfer fluids will promote an earlier boiling incipience and will improve the boiling HTC. The main mechanisms for the heat-transfer enhancement are the surfactant adsorption/desorption action at the fluid–vapor interface, surface tension, increased number of nucleation sites, and the improved wettability of the heating surface. Also, the addition of polymers (Section 3) may improve the HTC, which is most likely due to the decreased bubble coalescence and increased bubble nucleation.
(xiv) The self-rewetting fluids (Section 4) heat transfer benefits originate from their Marangoni convection, the capillarity effect, and the surface tension. The increase in the concentration of the self-rewetting fluids will improve the thermal performance of the fluid in comparison to water. However, the ideal concentration value remains incompletely clarified, given that the growing concentration may ameliorate the wettability but may degrade the surface tension and thermophysical properties of the operating fluid.
(xv) The nanofluids (Section 5) offer enhancing trends on the CHF, which originated in the improved surface wettability through the nanoparticle deposition during the pool boiling. However, there controversial findings remain related to the impact of nanofluids on the pool boiling heat-transfer behavior derived from parameters like imposed heat fluxes, base fluid type, the nature, size, shape, and concentration of the nanoparticles, and the synthesis and proof of the quality methods. Also, the stability of the dispersion of nanoparticles over time can be seriously affected by their clustering, sedimentation, and deposition onto the heating surface during boiling. Moreover, there are problems associated with the synthesis methods of these innovative fluids like complexities in the procedures and needed equipment, high investment cost, and hindered large scalability. Nonetheless, it can be noted that the nanofluids are very promising technological solutions for pool boiling heat-transfer enhancement.
(xvi) Regardless of the followed pool boiling heat-transfer enhancement approach, the pressure of the system is one factor that must be taken seriously in any pool boiling scenario. One of the main reasons for this comes from the fact that the pressure usually affects the thermophysical properties of the heat-transfer fluid, including the saturation temperature, latent heat of vaporization, and specific volume, which may have impacts on the bubble nucleation and detachment from the boiling surface; therefore, hey may affect the boiling thermal resistance. Also, a major part of the published results suggested that increasing pressures will increase the pool boiling heat-transfer enhancement.
(xvii) In general, there remains significant pool-boiling-related knowledge that should be fully explored for future improvements. Hence, extra pool boiling enhancement theoretical and empirical formulas, prediction models, and numerical simulations closely linked with enhanced boiling surfaces and innovative working fluids are highly recommended; such investigations will enable us to attain a better understanding of the underlying mechanisms and influencing factors of pool boiling enhancement methodologies. Also, to prepare modified surfaces for the pool boiling heat-transfer ability improvement of the systems, more interdisciplinary research studies should be conducted from fields such as material science and manufacturing engineering.

## Data Availability

Data sharing is not applicable.
